# Beyond the MHC: A canine model of dermatomyositis shows a complex pattern of genetic risk involving novel loci

**DOI:** 10.1371/journal.pgen.1006604

**Published:** 2017-02-03

**Authors:** Jacquelyn M. Evans, Rooksana E. Noorai, Kate L. Tsai, Alison N. Starr-Moss, Cody M. Hill, Kendall J. Anderson, Thomas R. Famula, Leigh Anne Clark

**Affiliations:** 1 Department of Genetics and Biochemistry, Clemson University, Clemson, South Carolina, United States of America; 2 Genomics and Computational Laboratory, Clemson University, Clemson, South Carolina, United States of America; 3 Department of Animal Science, University of California, Davis, California, United States of America; Stanford University School of Medicine, UNITED STATES

## Abstract

Juvenile dermatomyositis (JDM) is a chronic inflammatory myopathy and vasculopathy driven by genetic and environmental influences. Here, we investigated the genetic underpinnings of an analogous, spontaneous disease of dogs also termed dermatomyositis (DMS). As in JDM, we observed a significant association with a haplotype of the major histocompatibility complex (MHC) (DLA-DRB1*002:01/-DQA1*009:01/-DQB1*001:*01*), particularly in homozygosity (*P*-val = 0.0001). However, the high incidence of the haplotype among healthy dogs indicated that additional genetic risk factors are likely involved in disease progression. We conducted genome-wide association studies in two modern breeds having common ancestry and detected strong associations with novel loci on canine chromosomes 10 (*P*-val = 2.3X10^-12^) and 31 (*P*-val = 3.95X10^-8^). Through whole genome resequencing, we identified primary candidate polymorphisms in conserved regions of *PAN2* (encoding p.Arg492Cys) and *MAP3K7CL* (c.383_392ACTCCACAAA>GACT) on chromosomes 10 and 31, respectively. Analyses of these polymorphisms and the MHC haplotypes revealed that nine of 27 genotypic combinations confer high or moderate probability of disease and explain 93% of cases studied. The pattern of disease risk across *PAN2* and *MAP3K7CL* genotypes provided clear evidence for a significant epistatic foundation for this disease, a risk further impacted by MHC haplotypes. We also observed a genotype-phenotype correlation wherein an earlier age of onset is correlated with an increased number of risk alleles at *PAN2* and *MAP3K7CL*. High frequencies of multiple genetic risk factors are unique to affected breeds and likely arose coincident with artificial selection for desirable phenotypes. Described herein is the first three-locus association with a complex canine disease and two novel loci that provide targets for exploration in JDM and related immunological dysfunction.

## Author summary

Juvenile dermatomyositis (JDM) is an autoimmune disease of the skin and muscle influenced by both genetic and environmental components. Although genes independent of the MHC are thought to contribute to disease pathogenesis, their identification has been complicated by a paucity of biological samples, disease heterogeneity, and genetically diverse subjects. A naturally occurring inflammatory disease of domestic dogs, also termed dermatomyositis (DMS), is analogous to JDM and is the only animal model available for genetic study. We first determined that, as in JDM, a particular MHC haplotype confers susceptibility to DMS. Capitalizing on the genetic isolation of dog breeds and extremely low MHC diversity within affected breeds, we used multibreed genome-wide association studies to identify two novel loci. Through genome resequencing and additional genotyping, we uncovered highly associated polymorphisms in conserved positions of *PAN2* and *MAP3K7CL*. Analysis of three-locus genotypes revealed uniquely high frequencies among affected breeds and nine allelic combinations that confer moderate or high risk for DMS. The pattern of disease probability illustrates the presence of gene-gene interaction, as well as an inverse correlation between age of onset and number of risk alleles. This study highlights the utility of canine models for mapping susceptibility loci in complex diseases and detecting patterns of genetic interactions obscured in diverse human populations.

## Introduction

Juvenile dermatomyositis (JDM) is an autoimmune vasculopathy that causes a characteristic skin rash (heliotrope rash across the eyelids and Gottron’s papules on the bony prominences) and proximal muscle weakness [1]. It is the most frequently diagnosed childhood inflammatory myopathy, comprising 80% of all cases [1] and affecting 3.2 in every million children between the ages of 2 and 17 within the USA [2]. Prognosis is positively correlated with early diagnosis and swift treatment with corticosteroids and/or immunosuppressants [1,3]. While treatment of JDM is much improved overall, many children still suffer from chronic flare-ups [1].

Though the etiology is unknown, JDM is thought to be triggered by exposure to a virus or other environmental agent. Manlhiot et al. [4] reported that 71% of JDM patients had a clinical history consistent with infection prior to symptoms. Investigations into the class II major histocompatibility complex (MHC), *TNF*, and *IL1* identified several susceptibility and protective alleles, but their collective contribution to pathogenesis is poorly understood [5–8]. Recent genome-wide association studies (GWASs) to identify additional susceptibility loci in JDM confirmed a strong association with the MHC but failed to detect novel major risk factors, likely because of a paucity of biological samples and genetically heterogeneous populations [9,10].

In domestic dogs, an inflammatory vasculopathy of the skin and muscle, also termed dermatomyositis (DMS), is clinically, histologically, and immunologically similar to JDM and provides the only animal model available to study genetic risk factors [11–16]. The earliest clinical signs of DMS are crusting and scaling on the face, ears, tail tip, and/or across the bony prominences of the limbs and feet [17–19] (S1 Fig). Alopecia and more extensive skin lesions may develop over time, resulting in dermal scarring associated with erythema and mottled pigmentation [17,19]. Lesions persist for weeks to months, and may or may not chronically recur [17]. Muscle wasting manifests as atrophy of the head musculature; difficulty eating, drinking, and swallowing; and an atypical, high-stepping gait [17,19].

Similar to JDM, DMS is an immune-mediated disease [13,18,20] that typically develops following an environmental trigger, such as vaccination or viral infection, and is exacerbated by subsequent stressors like exposure to UV light [13,17,21,22]. Anecdotal reports indicate that rabies vaccination, parvovirus infection, owner surrender, or mistreatment commonly precede disease onset. Consistent with an environmental trigger, age at onset is variable with many cases occurring between seven weeks and six months of age, but others not developing until well into adulthood [17–19,23].

DMS is diagnosed almost exclusively in the genetically [24] and phenotypically similar collie and Shetland sheepdog breeds, suggesting the presence of a strong heritable component(s) arising from ancestors common to both breeds. A 1980s study of disease transmission in the collie eliminated simple Mendelian modes of inheritance [14]. In two test matings, an affected male collie sired litters from an affected collie and a healthy Labrador retriever. All six collie puppies were affected with variable degrees of severity, while three of the four mixed breed puppies developed milder forms of DMS. Retrospective pedigree analyses of the collie sire and dam showed a complete absence of affected ancestors [14].

The availability of a naturally-occurring canine model provides a new opportunity for the identification of genetic risk factors of JDM. The conserved genomic backgrounds of genetically isolated dog breeds have enabled detection of risk loci in complex diseases that are often obscured by heterogeneity within human cohorts [25–30]. Here, we evaluated class II MHC haplotypes, performed multibreed GWASs, and generated whole genome and transcriptome sequencing data to dissect the genetic basis of DMS. We uncovered common polymorphisms of the MHC and two novel loci that are strongly associated with DMS, as well as patterns of allelic inheritance that explain 93% of cases studied. A genetic test is now available to determine the likelihood of a dog developing DMS and to facilitate breeding decisions that avoid progeny having high-risk genotypes.

## Results and discussion

### Association of a major histocompatibility complex haplotype

Given the involvement of MHC genes in JDM, we first determined alleles of the highly polymorphic canine MHC class II dog leukocyte antigen (DLA) genes: *DLA-DRB1*, -*DQA1*, and -*DQB1*. Two locus (*DLA-DRB1* and *-DQB1*) and three locus (*DLA-DRB1*, *-DQA1*, and *-DQB1*) haplotypes were first generated for 50 collies and 117 Shetland sheepdogs, respectively. Because all observed haplotypes contained a unique *DLA-DRB1* allele, the 355 remaining dogs were genotyped for this locus only and the haplotype was inferred (Table 1).

**Table 1:** Frequency of *DLA-DRB1*/*-DQA1*/*-DQB1* haplotypes in collies and Shetland sheepdogs.

**COLLIE**					
DLA-DRB1*/-DQA1*/-DQB1*	**Cases (2n = 80)**	**Controls (2n = 370)**	**OR**	**95% CI**	***P*-value**
*002*:*01/009*:*01/001*:*01*	74	353	0.59	0.23–1.56	0.40
*006*:*01/050*:*11/007*:*01*	0	3	-	-	-
*015*:*01/009*:*01/001*:*01*	6	14	2.06	0.77–5.54	0.23
**SHETLAND SHEEPDOG**					
DLA-DRB1*/-DQA1*/-DQB1*	**Cases (2n = 184)**	**Controls (2n = 410)**	**OR**	**95% CI**	***P*-value**
*002*:*01/009*:*01/001*:*01* ^†^	158	301	**2.20**	**1.38–3.52**	**0.0010**
*015*:*01/009*:*01/001*:*01*	1	6	0.37	0.04–3.08	0.4454
*023*:*01/003*:*01/005*:*01*	24	102	**0.45**	**0.28–0.73**	**0.0011**
Other haplotypes	1	1	-	-	-

The number of times each haplotype was observed is reported for cases and controls with odds ratios (OR), 95% confidence intervals, and Fisher’s exact two-tailed *P*-values. Significant statistics are bolded.

^†^71 out of 92 cases and 109 out of 205 controls were homozygous for the *002*:*01/009*:*01/001*:*01* haplotype (OR = 2.98, 95% CI = 1.70–5.21, *P*-val = 0.0001).

We observed remarkably low DLA diversity among collies, with only three haplotypes present in 225 collies worldwide. This lack of heterogeneity precluded detection of associations with DMS, as 91% of collies were homozygous for the haplotype DLA-DRB1*002:01/-DQA1*009:01/-DQB1*001:*01*. In 297 Shetland sheepdogs, we identified two predominant haplotypes, *002*:*01/009*:*01/001*:*01* and *023*:*01/003*:*01/005*:*01*. The former was over-represented among cases (*P*-val = 0.0010, OR = 2.20), primarily because of increased homozygosity (*P*-val = 0.0001, OR = 2.98). We therefore conclude that *002*:*01/009*:*01/001*:*01* is a risk factor for DMS and that homozygosity confers increased susceptibility. Under the assumption that the causal alleles derive from an ancestor common to both breeds, we extrapolate the observed DLA risk to collies. The high frequency of the DLA risk haplotype in both populations indicates that additional loci must influence disease progression.

### Genome-wide association studies reveal signals on chromosomes 10 & 31

**Fig 1:**
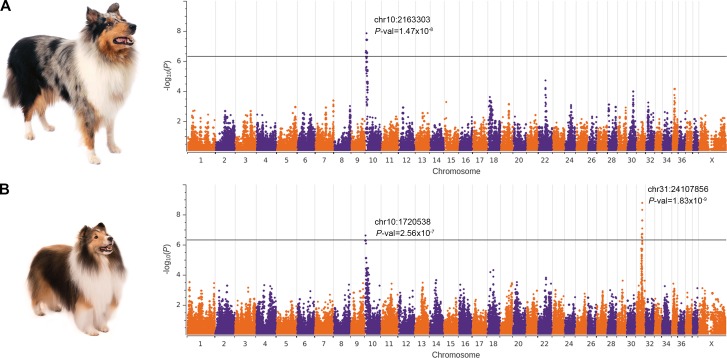
Manhattan plots of DMS GWASs in two breeds. (A) Collies: 31 cases and 23 controls. (B) Shetland sheepdogs: 66 cases and 45 controls. The –log_10_*P*-values (y-axis) for 98,520 Illumina SNPs are plotted against chromosome position (x-axis). The threshold for Bonferroni significance is shown as a black horizontal line. The *P*-value and position (canFam3) of the lead SNPs are reported.

We conducted an independent GWAS for each breed, using a total of 97 cases (31 collies, 66 Shetland sheepdogs), 68 controls (23 collies, 45 Shetland sheepdogs), and 98,520 SNPs after filtering. In collies, a single signal (*P*-val = 1.47X10^-8^) composed of 17 SNPs at the centromeric end of chromosome 10 exceeded Bonferroni significance (Fig 1A). In Shetland sheepdogs, this association was replicated (*P*-val = 2.56X10^-7^), and a second signal (*P*-val = 1.83X10^-9^) composed of 11 SNPs surpassing Bonferroni significance was detected on chromosome 31 (Fig 1B). Both associations persisted in a combined breed analysis (chr10: *P*-val = 2.3X10^-12^, chr31: *P*-val = 3.95X10^-8^) (S1 Table, S2 Fig); although the breeds possessed a common haplotype on chromosome 31, the Shetland sheepdogs appeared to drive this association. No associated SNPs were detected near the MHC loci on chromosome 12, likely a result of high homogeneity in our cohort and poor SNP coverage on the array [25].

**Fig 2:**
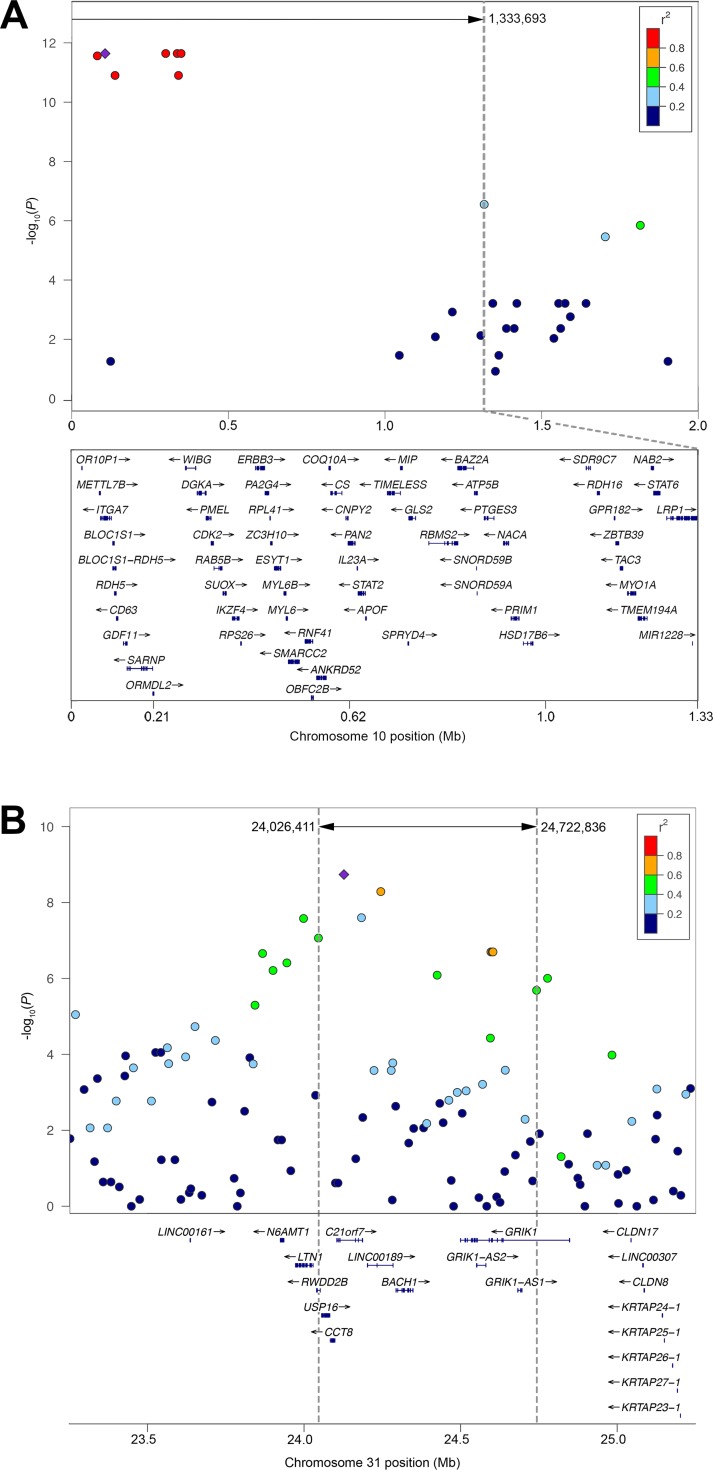
Regional plots depicting LD at two associated loci. Association results (-log_10_*P*) for (A) combined breed GWAS on chromosome 10 and (B) Shetland sheepdog GWAS on chromosome 31 are color-coded based on pairwise LD (r^2^) with the lead SNPs (purple diamond). Dotted lines demarcate the candidate interval. Genes within the region (based on hg18) are shown below the plot with chromosome positions reported in canFam3. On chromosome 10, the gene track is zoomed in to represent the candidate interval only; 4 genes were omitted from the figure (*DNAJC14*, *LOC440104*, *MMP19*, and *SLC39A5*).

On chromosome 10, 97% of all affected dogs were homozygous or heterozygous for the risk alleles of the lead SNPs. On chromosome 31, 88% of affected Shetland sheepdogs, but only 39% of affected collies, shared the risk alleles of the lead SNPs. As neither locus appeared to be independently necessary for disease development, we surveyed the extent of regional linkage disequilibrium (LD) to demarcate candidate intervals of ~1.33 Mb on chromosome 10 (Fig 2A) and ~696 kb on chromosome 31 (Fig 2B), harboring ~65 and 6 genes, respectively. The large size of the chromosome 10 region is attributed to lower recombination rates near the centromere and a dearth of informative SNPs.

### Identification of candidate variants in *PAN2* and *MAP3K7CL*

Whole genome resequencing was performed for four affected dogs (three collies and one Shetland sheepdog) and two unaffected collies, resulting in 17X to 41X coverage. Variants were filtered for those lying within our delineated intervals (chr10:1–1,333,693; chr31:24,026,411–24,722,836) and following the inheritance pattern of the most significantly associated SNPs in the affected dogs (S2 and S3 Tables). Five intergenic and two intronic variants were unique to these breeds (*i*.*e*., not present in the Boxer reference genome, dbSNP, or 27 whole genome sequences from 16 other breeds); however, most were in repetitive regions and none were in conserved positions. Thus, the pathogenic variants were likely to be common polymorphisms, so we next prioritized variants within predicted exons and splice sites of genes expressed in skin for further study.

To confirm exon/intron boundaries predicted by Ensembl 79 and establish expression of variants in affected tissue, we generated RNAseq data. We obtained a minimum of 89 million reads per tissue from active skin lesions of two affected dogs (one collie and one Shetland sheepdog) and skin from one unaffected Australian shepherd dog. All genes expressed in skin within the candidate regions were also expressed in affected tissues. Seventeen exonic variants were expressed; seven of these were nonsynonymous and evaluated using *in silico* programs [31–33] (Table 2).

**Table 2:** Expressed exonic changes on chromosomes 10 and 31.

Position	Gene	A1/A2	AA change	Poly-Phen2	PANTHER	SIFT	*P*-value
**CHROMOSOME 10**							**Cases n = 93 Controls n = 63**
95042	*ITGA7*	T/C	Val/Val	-	-	-	-
121835	*RDH5*	G/A	Ala/Ala	-	-	-	-
222809	*MMP19*	T/G	Gln/Pro	0	91	0.43	-
331135	*SUOX*	A/G	His/Arg	0	85	N/A	-
**565958**	***ANKRD52***	**G/C**	**Ser/Cys**	**0.92**	**361**	**0.02**	**1.93X10**^**-17**^
**627760**	***PAN2***	**G/A**	**Arg/Cys**	**1**	**1628**	**0**	**1.93X10**^**-17**^
1127082	*RDH16*	C/T	Ala/Ala	-	-	-	-
1228277	*NAB2*	G/C	Gly/Arg	0	85	N/A	-
1239562	*STAT6*	G/A	Thr/Met	0.98	361	0.05	1.52X10^-4^
1286150	*LRP1*	C/T	Asn/Asn	-	-	-	-
1333693	*LRP1*	G/A	Gln/Gln	-	-	-	-
**CHROMOSOME 31**							**Cases n = 63 Controls n = 45**
24068039	*CCT8*	T/C	Ala/Ala	-	-	-	-
**24132273**	***MAP3K7CL***	**Indel/-**	**5′ UTR**	**N/A**	**N/A**	**N/A**	**2.09X10**^**-11**^
24132343	*MAP3K7CL*	A/C	5′ UTR	N/A	N/A	N/A	1
24292521	*BACH1*	A/G	Asn/Asp	0.007	324	0.21	-
24294659	*BACH1*	A/G	Glu/Glu	-	-	-	-
24295208	*BACH1*	A/G	Pro/Pro	-	-	-	-

Alleles 1 (A1 = minor allele) and 2 (A2 = reference allele) are reported. Amino acid changes are based on the dog reference genome. *P*-values are reported for matched populations. Variants with PolyPhen2 scores ranging from 0.85–1, PANTHER preservation time (in millions of years) >450my, and SIFT scores ranging from 0–0.05 are considered deleterious/probably damaging. Variants more strongly associated with DMS than the most associated SNPs from the array are bolded.

We genotyped a subset of our mapping population for three nonsynonymous SNPs on chromosome 10 (*ANKRD52* g.565958G>C, *PAN2* g.627760G>A, and *STAT6* g.1239562G>A) that were predicted to be deleterious or probably damaging by more than one *in silico* program. The *ANKRD52* and *PAN2* variants were more strongly associated with DMS than the lead SNP. These variants were in perfect linkage disequilibrium with each other; however, *PAN2* g.627760G>A was assigned damaging scores with higher confidence by *in silico* programs (Table 2). We therefore focused further studies on *PAN2* g.627760G>A, encoding p.Arg492Cys (XP_531635.3), although *ANKRD52* cannot be excluded. On chromosome 31, we genotyped Shetland sheepdogs for a SNP (g.24132343A>C) and an indel (c.383_392ACTCCACAAA>GACT, XM_846337.4), both located in a 5′ non-coding exon of *MAP3K7CL*. Only the indel was associated with DMS (Table 2, S3 Fig). In an expanded, combined population (132 affected and 390 unaffected collies and Shetland sheepdogs), both the *PAN2* (*P*-val = 2.08X10^-35^) and *MAP3K7CL* (*P*-val = 1.45X10^-33^) variants were highly associated with DMS (S4 Table).

*PAN2* (or *USP52*) encodes the catalytic subunit of the poly(A) nuclease deadenylation complex (PAN2-PAN3) and is one of two exonucleases involved in mRNA degradation in eukaryotes [34,35]. Deadenylation plays a role in translational regulation of inflammatory response [36]. Independent of this function, PAN2 also stabilizes *HIF1A* transcripts via their 3*′*-UTR, which contain AU-rich elements (AREs), and may be involved in regulating other transcripts having AREs [37]. *HIF1A*, a key regulator of inflammation [38], and other ARE-containing transcripts, such as *IL-6* [39], are upregulated in JDM [40, 41]. *PAN2* is widely expressed and highly evolutionarily conserved [35]; human (NP_001120932.1) and dog (XP_013972628.1) amino acid sequences share 98% identity.

*MAP3K7CL* (also known as *TAK1L* or *C21orf7*) is a poorly studied kinase gene that is transcriptionally active in immunological tissues and expressed primarily in peripheral blood leukocytes [42,43]. Human (NP_001273546.1) and dog (XP_013965340.1) MAP3K7CL protein sequences share >90% identity. The transcription factors RUNX3 and EP300 bind the 5′ non-coding exon of human *MAP3K7CL* (UCSC Genome Browser ENCODE Transcription Factor ChIP-seq track). In this exon, the c.383_392ACTCCACAAA>GACT indel causes the loss of six conserved base pairs, omitting a RUNX3 binding motif (*P*-val = 2.07X10^-3^ from TOMTOM [44]) (S3 Fig). RUNX3 has known roles in inflammatory response (*e*.*g*., thymopoiesis [45,46] and the TGF-β signaling cascade [47]), and it has been directly implicated in a number of immune-related diseases [48–50]. Furthermore, SNPs disrupting RUNX binding motifs in target genes confer susceptibility to autoimmune rheumatic diseases, including psoriasis [51] and systemic lupus erythematosus [52].

### Analysis of three-locus genotypes reveal gene-gene interactions

**Fig 3:**
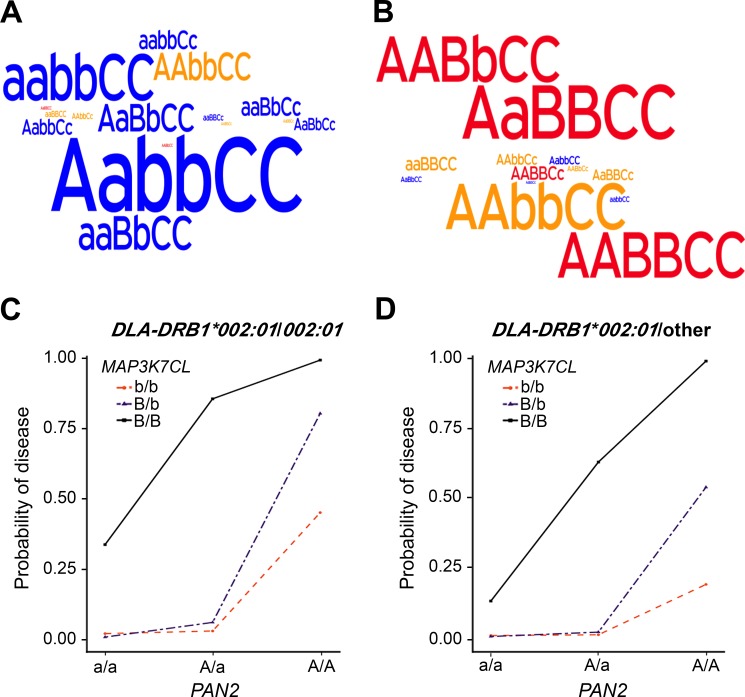
Three-locus genotype disease probabilities and frequencies (132 cases, 390 controls). Word clouds of three-locus genotypes illustrate frequency differences between (A) controls and (B) cases with high-risk genotypes in red, moderate-risk in yellow, and low-risk in blue. Probability of disease (y-axis) for all combinations of *PAN2* (x-axis) and *MAP3K7CL* genotypes are plotted in dogs (C) homozygous and (D) heterozygous for DLA-DRB1*002:*01*.

We next considered three-locus genotypes in our expanded, combined population (132 affected and 390 unaffected dogs) where *A* = the *PAN2* variant encoding p.Arg492Cys, *B* = *MAP3K7CL* c.383_392ACTCCACAAA>GACT, and *C* = DLA-DRB1*002:*01*, lower-case letters denote wild type alleles (*c* represents any alternate allele of *DLA-DRB1*). Only 4% of dogs possessed a three-locus genotype with *cc*, barring further analysis of those nine genotypes. We considered nine of the remaining genotypes to be low-risk, as less than 6% of dogs with these allelic combinations had DMS (Table 3). Among healthy dogs (Fig 3A), the most frequently observed genotypes were *AabbCC* (24%) and *aabbCC* (15%). Based on penetrance, we classified five genotypes as conferring moderate risk (33–50%) and four as high risk (90–100%) for DMS. All cases possessed at least two risk alleles and all but one were homozygous for at least one risk allele. The most common genotypes of affected dogs (Fig 3B) were *AaBBCC* (20% of cases), followed by *AAbbCC*, *AABbCC*, and *AABBCC* (17% of cases each).

**Table 3:** Distribution of three-locus genotypes in 132 cases and 390 controls with penetrance, 95% confidence intervals, and *P*-values.

Genotype	Collies		Shetland sheepdogs		Combined			
	Cases (n = 40)	Controls (n = 185)	Cases (n = 92)	Controls (n = 205)	Penetrance (%)	Risk*	95% Confidence Interval	*P*-value**
*aabbcc*	0	1	0	1	-	-	0–0.842	1
*aabbCc*	0	5	0	18	-	low	0–0.148	**0.0023**
*aabbCC*	1	43	1	17	-	low	0.004–0.112	**0**
*Aabbcc*	0	0	0	3	-	-	0–0.708	0.577
*AabbCc*	0	3	1	15	-	low	0.001–0.260	0.060
*AabbCC*	2	71	2	23	-	low	0.011–0.101	**0**
*aaBbcc*	0	0	0	5	-	low	0–0.522	0.399
*aaBbCc*	0	1	0	21	-	low	0–0.154	**0.002**
*aaBbCC*	0	10	0	32	-	low	0.084	**0**
*AaBbcc*	0	0	0	1	-	-	0–0.975	1
*AaBbCc*	0	0	0	14	-	low	0–0.232	**0.028**
*AaBbCC*	0	13	2	23	-	low	0.006–0.177	**0.002**
*AAbbcc*	1	0	0	0	-	-	0.025–1	0.253
*AAbbCc*	4	6	1	1	42	moderate	0.152–0.723	0.194
*AAbbCC*	15	31	7	3	39	moderate	0.265–0.532	**0.021**
*aaBBcc*	0	0	0	1	-	-	0–0.975	1
*aaBBCc*	0	0	0	6	-	low	0.0459	0.348
*aaBBCC*	0	0	6	7	46	moderate	0.192–0.749	0.107
*AaBBcc*	0	0	2	1	-	-	0.094–0.992	0.160
*AaBBCc*	0	0	5	5	50	moderate	0.187–0.813	0.136
*AaBBCC*	3	0	23	3	90	high	0.762–0.978	**0**
*AABbcc*	0	0	3	0	-	-	0.292–1	**0.016**
*AABbCc*	0	0	2	4	33	moderate	0.043–0.777	0.647
*AABbCC*	11	1	11	1	92	high	0.730–0.990	**0**
*AABBcc*	0	0	0	0	-	-	-	-
*AABBCc*	0	0	7	0	100	high	0.590–1	**0.0001**
*AABBCC*	3	0	19	0	100	high	0.846–1	**0**

Significant values in bold.

*Risk interpretations were only made for three-locus genotypes observed at least five times.

**P-values are calculated for each genotype compared to the population as a whole (0.253, 132 affected dogs out of 522 total dogs).

Interestingly, only affected dogs possessed *AABBCc* or *AABBCC* (n = 29), indicating that DMS is fully penetrant in dogs having these combinations. Epistasis plots illustrated that genotypes with at least one *a* or *b* allele confer a lower probability of disease when a *c* allele is present, compared to their *CC* counterparts (Fig 3, compare 3C and 3D). The plots also depicted a greater probability of disease than expected under a strictly additive model, providing evidence for additive-by-additive epistasis between the chromosome 10 and 31 loci [53,54]. We noted at least one ARE in *MAP3K7CL*, presenting a mechanism for interaction with PAN2. No difference in gene interactions was observed between the sexes (S4 Fig).

Information regarding age at onset or diagnosis was available for 91 dogs. We compared dogs having two, three, or four risk alleles across *PAN2* and *MAP3K7CL* and observed an inverse correlation between age of onset and number of risk alleles (S5 Fig). Dogs having four risk alleles developed DMS at a significantly younger median age (5 months) than did dogs with only two risk alleles (18.5 months). The complete penetrance of *AABB* genotypes, combined with an early age of onset, suggest that these dogs may be hypersensitive to commonplace environmental stimuli (*e*.*g*., routine puppy vaccinations).

### Collies and Shetland sheepdogs have uniquely high frequencies of associated alleles

**Fig 4:**
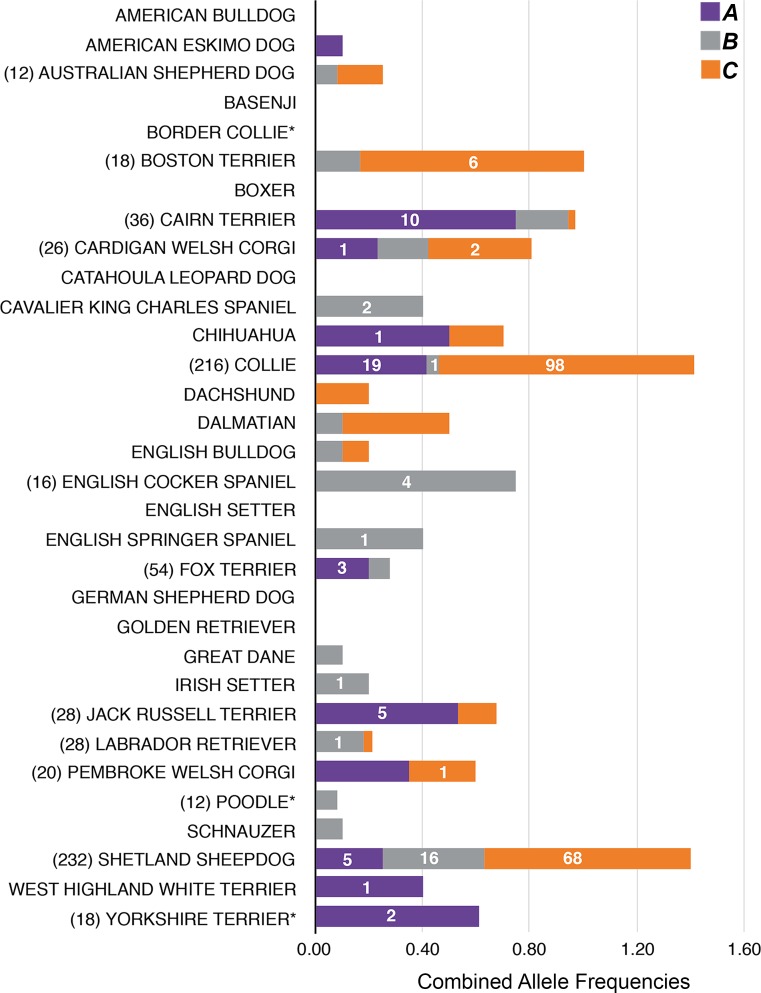
Frequencies of DMS-associated polymorphisms across breeds. The number of alleles used to calculate the frequencies is shown to the left of the breed name, if > 10. Risk allele frequencies (x-axis) for each gene are represented by a single, segmented colored bar: the *PAN2* variant encoding p.Arg492Cys is represented in purple, *MAP3K7CL* c.383_392ACTCCACAAA>GACT in gray, and DLA-DRB1*002:*01* in orange. The number of homozygous dogs observed is reported on the corresponding color bar. Asterisks denote breeds lacking DLA-DRB1*002:*01* in this study, but which were previously reported to possess the allele [57].

All three identified variants associated with DMS are polymorphisms present in several breeds, raising the question: why are other breeds rarely, if ever, affected by DMS? We genotyped five or more unrelated individuals from each of 30 diverse breeds for all three loci (Fig 4). The only other breeds to possess all three risk alleles were Cardigan Welsh corgis and Cairn terriers. Three Jack Russell terriers had moderate-risk genotypes (*AAbbCc*), as did one Cardigan Welsh corgi (*AABbCc*); both breeds are occasionally diagnosed with dermatomyositis-like disease [55,56]. None of the 229 individuals possessed a high-risk genotype (S5 Table). Interestingly, Labrador retrievers had both *B* and *C*, which could have enabled moderate or high risk genotypes (*AaBBCc* or *AaBBCC*) in puppies from the outcross mating described by Haupt et al. [14].

Combined frequencies of risk alleles in other breeds were dramatically lower than those observed among collie and Shetland sheepdog populations, and homozygosity for a risk allele (a characteristic of all moderate- to high-risk genotypes) was rare. Additionally, breeds having a high frequency of one risk allele had few or no risk alleles at the other loci. For example, Cairn terriers had a high frequency of *A* (75%) but low frequencies of *B* (19%) and *C* (3%), and no high- or moderate-risk genotypes were observed among 18 individuals. These findings suggest that independently the polymorphisms are neither deleterious nor selected against.

It is likely that recent artificial selection for phenotypes shared by collies and Shetland sheepdogs led to increased frequencies of *A*. We propose that persistence of *A* in these two breeds is attributed to linkage disequilibrium (D′ = 0.998) between wildtype *PAN2* (*a*) and another chromosome 10 allele, *Merle* of *PMEL*. In heterozygosity, *Merle* causes a popular pigmentation pattern (see collie in Fig 1A), but homozygosity for the allele results in severe hypopigmentation often with auditory and ocular defects [58]. Wildtype *PMEL* occurred on chromosomes with either *A* or *a*, whereas *Merle* was only found in conjunction with *a*. Accordingly, the *Merle* phenotype was underrepresented in affected dogs (*P*-value = 0.0018), 64% of which were homozygous for *A*. Consistent selection for heterozygosity (but not homozygosity) for *Merle* would simultaneously encourage maintenance of both *PAN2* alleles. To our knowledge, there are no loci on chromosome 31 under positive selection for maintenance of a characteristic phenotype of collies and/or Shetland sheepdogs.

Across five collie genomes, we observed a 1.2 Mb selective sweep on chromosome 12 (S6 Fig) encompassing the MHC class II loci and leading to near fixation of the *002*:*01/009*:*01/001*:*01* haplotype. We suggest that essentially all purebred collies have increased susceptibility for DMS, conferred by homozygosity for allele *C*. The Shetland sheepdog population has retained a less common second haplotype that permits heterozygosity at the DLA loci, associated with a lower risk for developing DMS. Ironically, this reduced risk may have masked the presence of otherwise high-risk genotypes (*i*.*e*., *AABb* and *AaBB*) and hindered selection against *A* and *B* alleles.

We observed striking allele frequency differences between the two affected breeds at *PAN2* and *MAP3K7CL*: collies had a higher frequency of *A*, 42% (25% in Shetland sheepdogs), whereas Shetland sheepdogs had a higher frequency of *B*, 38% (5% in collies) (Fig 4). Consequently, the frequency of observed allelic combinations varied between the breeds. The most common genotypes in healthy dogs were *aaBbCC* in Shetland sheepdogs, 16% (5% in collies), and *AabbCC* in collies, 38% (11% in Shetland sheepdogs). Among affected dogs, *AaBBCC* predominated in Shetland sheepdogs, 25% (8% in collies), whereas *AAbbCC* was the most frequent genotype in diseased collies, 38% (8% in Shetland sheepdogs). The latter finding is interesting given that *AAbbCC* is only a moderate-risk genotype. Among collies having this genotype, 67% were unaffected by age 8, whereas only 30% of Shetland sheepdogs with this genotype were disease-free. This discrepancy in disease probabilities between breeds was unique to this genotype. Further studies will be necessary to determine if other loci confer additional risk for or protection from DMS.

The contribution of alleles from multiple loci explains the spontaneous appearance of the disease in lines with no prior history [14] and has hindered elimination of DMS in the absence of a genetic test. For example, a mating between two healthy dogs having low risk genotypes (*e*.*g*., *AaBbCC* x *AaBbCC*) can produce puppies with low, moderate, or high risk for DMS. This study has led to the first three-locus genetic test for a complex disease of dogs, which will allow breeders to carefully reduce the frequency of *A* and *B* among collie and Shetland sheepdog populations while preserving genetic diversity and desirable breed characteristics.

In a canine model of JDM, we have identified a complex pattern of causation involving three independent loci, two of which offer new targets for exploration in JDM. Furthermore, these data provide support for the involvement of genetic risk factors independent of the MHC in human inflammatory myopathies. While further experiments are necessary to determine the exact contribution of the chromosome 10 and 31 loci, our findings suggest that DMS may result from an inability to properly regulate inflammatory response. This work highlights the utility of the canine model for unraveling genetic susceptibility conferred by common polymorphisms and/or gene-gene interactions in complex diseases.

## Methods

### Ethics statement

All samples were obtained with informed consent according to protocols approved by the Clemson University Institutional Review Board (IBC2008-17) and IACUC (2012–039).

#### Study population

Three populations were assembled: DMS-affected dogs (92 Shetland sheepdogs, 40 collies), control dogs for GWAS (45 Shetland sheepdogs, 23 collies), and unaffected dogs (160 Shetland sheepdogs, 162 collies). Affected dogs were diagnosed either through histopathology (75 Shetland sheepdogs, 30 collies) or by a veterinarian based on clinical signs and elimination of demodectic mange, a differential diagnosis. Pedigrees were collected when available; some samples were obtained from affected dogs surrendered to rescue organizations without paperwork. Twenty percent of affected dogs were collected internationally, and in each case a regionally-matched control sample was obtained.

Control dogs for GWAS were eight years of age or older at time of collection, had no known family history of DMS, had no personal history of skin disease, and were unrelated to other study participants within two (most often three) generations. Pedigrees were obtained from all control dogs. The population of unaffected dogs had no clinical signs of DMS and were eight years of age or older at time of collection. This subset was collected without regard to family history of DMS, presence of other skin disorders, or relationship to other study participants. Archival samples from 229 dogs of 30 other breeds were not closely related to each other to our knowledge.

Whole blood or buccal cells were obtained from each dog, and genomic DNA was isolated according to the Puregene DNA Isolation protocol (Gentra). Skin punch biopsies from active lesions or healthy tissue were also obtained from one Shetland sheepdog, one collie, and one Australian shepherd dog and preserved in RNAlater (Ambion).

#### DLA class II genotyping

The hypervariable regions of *DLA-DRB1*, *-DQA1* and *-DQB1* were sequenced and genotyped according to protocols previously described [59–61]. *DLA-DQA1* was largely uninformative in collies and was inferred. Association of a haplotype or allele state with DMS was assessed through Fisher’s exact tests, using VassarStats (Web Site for Statistical Computation, Vassar College, Poughkeepsie, NY).

#### Genome-wide association and LD analyses

Genotyping was performed for 166 dogs (85 males and 81 females) using the Illumina CanineHD BeadChip, containing 173,662 SNPs. One sample having call rates <95% was excluded. SNPs having call rates <95%, minor allele frequencies <5%, and/or significant deviation from Hardy-Weinberg equilibrium (*P*-value <0.0001) in the control dogs were excluded. No evidence of genomic inflation was observed in the combined Shetland sheepdog and collie analysis (λ = 1.04). Fisher’s exact *P*-values were calculated under a dominant model.

LD pairwise analysis was performed to calculate r^2^ values, which were plotted using LocusZoom [62]. We calculated r^2^ values between SNPs on chromosome 10 using all controls. For chromosome 31, r^2^ values were calculated using only control Shetland sheepdogs because no association was detected in collies alone. Assuming that the pathogenic variant would be in high LD with the lead SNP, candidate regions were characterized by SNPs with pairwise r^2^ ≥ 0.6 and defined by the first flanking associated SNPs in lower LD. Filtering and statistical analyses were conducted with SNP & Variation Suite v8 (SVS, Golden Helix). All chromosome positions throughout the text are reported in CanFam3.1.

#### Whole genome resequencing

Three affected and two control collies were selected for whole genome resequencing along with one affected Shetland sheepdog. Genomic DNA fragments of approximately 500bp were gel size selected for each sample and sequenced on two lanes of an Illumina HiSeq 2000, generating 2x100 (collies) and 2x125 bp (Shetland sheepdog) paired-end reads. 531 to 997 million total reads were generated for the five collies. Over one billion total reads were generated for the Shetland sheepdog. Paired reads were aligned to the indexed reference (CanFam3.1) with Bowtie2 [63] under sensitive parameters. The alignments were sorted and indexed with SAMtools [64,65] to be visualized in the Interactive Genomics Viewer [66].

#### RNA isolation and sequencing

Total RNA was extracted from 30-40mg of skin punch biopsy tissue from active lesions (collie and Shetland sheepdog) or healthy skin (Australian shepherd) using the ToTALLY RNA kit (Ambion), according to the manufacturer’s protocols. RNA samples were treated with DNase to remove DNA contamination, using the DNA-free kit (Ambion). Samples were quantitated on a NanoDrop 1000 spectrophotometer (Fisher Scientific).

Three RNAseq libraries were constructed per dog using normalized total RNA and the manufacturer's protocol for one of the following: TruSeq RNA library prep kit v2.0 (Illumina) or TruSeq stranded total RNA library prep kit (Illumina). An Agilent Bioanalyzer 2100 was used for size validation. Each sample was sequenced at 2x125 bp paired-end on an Illimina HiSeq 2500 to a depth of at least 22 million reads.

FastQC from the Babraham Institute was used to assess read quality before and after preprocessing by Trimmomatic [67], which removed low quality bases and remaining sequence adapters. Trimmed reads for each sample were aligned to CanFam3.1 using gsnap [68], and SAMtools [64,65] was used to generate sorted and indexed bam files.

#### Variant filtering and genotyping

SAMtools and BCFtools [64,65] were used to generate variant call files for each sample, which were analyzed in SVS. On chromosome 10 (1–1,333,693 bp) variants that were heterozygous in affected collies 1 & 2 and homozygous in affected collie 3 and the affected Shetland sheepdog (*i*.*e*., reference/reference or alternate/alternate), consistent with the allele states at the lead SNPs, were selected. On chromosome 31 (24,026,411–24,722,836 bp), all homozygous variants in the Shetland sheepdog were selected. Ensembl 79 was used to identify variants lying within predicted exons and 10 bp flanking sequences to capture splice sites. RNAseq data were manually inspected in IGV to determine whether predicted exonic variants were expressed in the affected dogs. Alternate variants were investigated using dbSNP and 27 genomes of 16 other breeds (either sequenced as part of ongoing studies or shared by other research groups) to determine whether any were unique to collies and Shetland sheepdogs. SIFT [31], PolyPhen [32], and PANTHER [33] were used to predict the impact of the amino acid substitutions. Genotyping of variants in additional dogs was accomplished by restriction digest assays or Sanger sequencing (S6 Table).

#### Age of onset

Because age is not distributed as a normal random variable, we made use of the Weibull distribution, a density commonly used for time-to-event data [69]. Our goal was to estimate the mean and median age of onset, along with their confidence intervals, to facilitate comparisons across the three genotypic groups.

Taking advantage of a Bayesian strategy, we assume y_ij_ ~ Weibull (r, $e^{{-b}_{i}}$), where y_ij_ is the observed age at diagnosis for the j-th (j = 1, 2, …, n_i_) dog in the i-th (i = 1,2, …,3) genotypic class and b_i_ is the effect of the i-th genotypic class. With this representation of the scale (r) and shape ($e^{{-b}_{i}}$) parameters of the Weibull density, the median of the i-th genotypic class is $e^{{-b}_{i}}$ log(2)^1/r^ and the mean of the i-th genotypic class is $e^{{-b}_{i}}$ Γ(1 + 1/r).

In addition, we assumed a prior density for the unknown genetic parameters (b_i_) to have a null mean and common variance σ (*i*.*e*., (b_i_ ~ N(0, σ)). We consider the weakly informative prior for σ ~ Cauchy(0,25) [70]. For the scale parameter, r, we assume the weakly informative prior of r ~ exponential(0.001). The Monte Carlo Markov Chain (MCMC) sampling process was run in 4 chains through the public-domain software Stan [71], with each chain based on 50,000 total samples, and the first 20,000 were removed as part of the warm-up process, then thinned to every 20th sample, resulting in a MCMC sample of 6,000 values [71]. Convergence to the posterior density was evaluated by the Gelman-Rubin test statistic, where values less than 1.05 indicate that the MCMC sampling process was adequate to the data evaluated [72].

#### Genetic interaction analyses

All affected (n = 132) and healthy (n = 390) dogs were used to investigate possible interactions between the *PAN2* variant encoding p.Arg492Cys and *MAP3K7CL* c.383_392ACTCCACAAA>GACT in dogs homozygous for *002*:*01/009*:*01/001*:*01* vs. dogs heterozygous for the haplotype. Three-locus genotypes observed in fewer than five dogs were excluded. For a given disease state (cases vs. controls) and a given pair of genotypic classes *i* (*i* = *A*/*A*, *A*/*a*, or *a*/*a*) and *j* (*j* = *B*/*B*, *B*/*b*, or *b*/*b*), n_cases ij_ ~ Binomial(n_cases ij_ + n_controls ij_, p_ij_) where n_cases ij_ is the number of observed cases in the combination of the genotypic class of locus *i* and that of locus *j*, n_controls ij_ is the number of unaffected dogs in the combination of genotypic class of locus *i* and that of locus *j*. Finally, p_ij_ is the probability of disease for the combined genotypic classes of *i* and *j*.

Estimation of the unknown elements of our model must ensure estimates of p_ij_ within the interval [0, 1], recognizing that several genotypic classes have zero or few cases. We utilized a hierarchical Bayesian framework facilitated through the public-domain software Stan [71] to address this problem [73]. Stan can be accessed through the public-domain language R [74]. Log-odds was used to estimate p_ij_, *i*.*e*., log(p_ij_ /(1- p_ij_)) = intercept + addA_i_ + domA_i_+ addB_j_ + domB_j_ + add x add_ij_ + add x dom_ij_ + dom x add_ij_ + dom x dom_ij_, where intercept represents a term common to all genotypic classes, addA_i_, addB_j_ domA_i_ and domB_j_ represent the additive and dominance terms respectively for loci *A* and *B*, and add x add_ij_ + add x dom_ij_ + dom x add_ij_ + dom x dom_ij_ represent the four possible epistatic interaction terms for all possible additive and dominance combinations [53].

We assumed a prior density for the intercept and unknown genetic parameters, with null mean and common variance σ (*i*.*e*., N(0, σ)). Subsequently, we consider the weakly informative prior for σ ~ Cauchy(0,25) [70]. We used the same MCMC parameters here as described above.

#### Chromosome 12 selective sweeps

We identified all SNPs on chromosome 12 present in five collie genomes (3 cases and 2 controls) and used a creeping window size ≤1 Mb to identify runs of homozygosity [75]. Windows containing fewer than 50 SNPs were excluded and gaps >10kb between SNPs were ignored. The heterozygosity (Hp) statistic was calculated for all windows and Z-transformed, making the average Hp value equal to zero and the standard deviation equal to 1. The–ZHp distribution was plotted in R to show putatively selected regions greater than 3.4 standard deviations from the mean.

#### PMEL

D' between *PMEL* and *PAN2* was calculated as a measure of LD using all dogs for which coat pattern phenotypes were available (112 cases, 385 controls). A total of 98 dogs (10 cases and 88 controls) were described as merle-patterned, a semi-dominant trait caused by the *Merle* (*M*) allele of *PMEL*, and assumed to possess the *Mm* genotype. Computation of D', along with a test of significance from zero, was facilitated through the package *genetics* [76], a public domain program that is part of the R language [74].

### Accession numbers

SNP data are available in dbSNP under BioProject number PRJNA338128. All whole genome and transcriptome data generated for this study were deposited in SRA genomes under accesssion number SRP081080. Accession numbers for eight of the 27 other breeds used in variant filtering are SRX1360633, SRX1360635, SRX1360637, SRX1360639, SRX1022256, SRX1022262, SRX1022286, and SRP081080.

## Supporting information

S1 Fig Clinical presentation of dermatomyositis. Canine dermatomyositis is a vasculopathy that initially manifests as cutaneous lesions across the bony prominences of the face, tail tip, limbs, and feet, shown here. Some dogs develop alopecia and more extensive lesions over time, resulting in dermal scarring associated with erythema and mottled pigmentation.
(PDF)

S2 Fig Manhattan and quantile-quantile plots of combined DMS GWAS (97 cases vs. 68 controls). The –log10*P*-values (y-axis) for 98,520 Illumina SNPs are plotted against chromosome position (x-axis). The threshold for Bonferroni significance is shown as a black horizontal line. The *P*-value and position (canFam3) of the lead SNPs are reported. The Q-Q plot is boxed in purple and plots observed vs. expected Fisher’s exact –log10*P*-values. The inflation factor (λ) is shown on the Q-Q plot.
(PDF)

S3 Fig *MAP3K7CL* indel with conservation and RUNX3 binding motif. UCSC 100 Vertebrates track for human chr21:29,130,846–29,130,860 showing the G insertion and seven base pair deletion created by *MAP3K7CL* indel (Dog c.383_392ACTCCACAAA>GACT). Bases in gray differ from the dog reference sequence. The canine sequence is highlighted in yellow. The RUNX3 binding motif is underlined.
(PDF)

S4 Fig Three-locus genotype disease probabilities by sex. Probability of disease (y-axis) for all combinations of *PAN2* (x-axis) and *MAP3K7CL* genotypes are plotted in dogs (73 affected and 145 unaffected males; 59 affected and 245 unaffected females) (top) homozygous and (bottom) heterozygous for DLA- DRB1*002:*01*.
(PDF)

S5 Fig Median age of onset for combinations of *PAN2* and *MAP3K7CL* genotypes. Median age of onset is plotted for genotypes consisting of 2 (AAbb, aaBB, AaBb), 3 (AABb, AaBB), and 4 (AABB) risk alleles at *PAN2* and *MAP3K7CL*. CC and Cc genotypes were combined for analyses. Number of individuals is shown to right.
(PDF)

S6 Fig Collie selective sweeps on chromosome 12. ZH(*p*) values for all creeping windows containing 50 or more SNPs are plotted against chromosome position. Creeping windows are ≤1 Mb.
(PDF)

S1 Table SNPs exceeding Bonferroni significance (5.08X10^-7^). (PDF)

S2 Table Chromosome 10 variants segregating with the lead SNPs in the affected dogs. (PDF)

S3 Table Chromosome 31 variants segregating with the lead SNPs in the affected Shetland sheepdog. (PDF)

S4 Table Frequency of *PAN2* and *MAP3K7CL* genotypes. (PDF)

S5 Table Three-locus genotypes for 229 individuals of 30 breeds. (PDF)

S6 Table Primers and genotyping method for variants. (PDF)
