# Supplementary material for: Beyond the MHC: A canine model of dermatomyositis shows a complex pattern of genetic risk involving novel loci
Source: PLoS Genet. 2017 Feb 3;13(2):e1006604. doi: 10.1371/journal.pgen.1006604 (PMC5315411; doi:10.1371/journal.pgen.1006604)
Supplement: S1 Fig — Canine dermatomyositis is a vasculopathy that initially manifests as cutaneous lesions across the bony prominences of the face, tail tip, limbs, and feet, shown here. Some dogs develop alopecia and more extensive lesions over time, resulting in dermal scarring associated with erythema and mottled pigmentation. (PDF) [file pgen.1006604.s001.pdf]

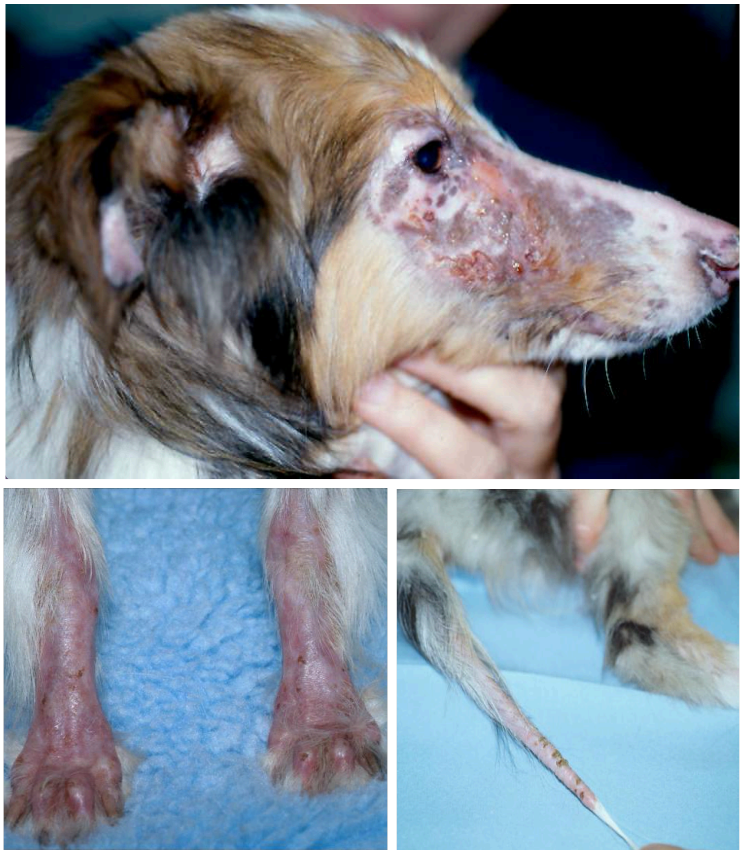

S1 Fig. Clinical presentation of dermatomyositis. Canine dermatomyositis is a vasculopathy that initially manifests as cutaneous lesions across the bony prominences of the face, tail tip, limbs, and feet, shown here. Some dogs develop alopecia and more extensive lesions over time, resulting in dermal scarring associated with erythema and mottled pigmentation.
