# Supplementary material for: Beyond the MHC: A canine model of dermatomyositis shows a complex pattern of genetic risk involving novel loci
Source: PLoS Genet. 2017 Feb 3;13(2):e1006604. doi: 10.1371/journal.pgen.1006604 (PMC5315411; doi:10.1371/journal.pgen.1006604)
Supplement: S2 Fig — The –log10P-values (y-axis) for 98,520 Illumina SNPs are plotted against chromosome position (x-axis). The threshold for Bonferroni significance is shown as a black horizontal line. The P-value and position (canFam3) of the lead SNPs are reported. The Q-Q plot is boxed in purple and plots observed vs. expected Fisher’s exact –log10P-values. The inflation factor (λ) is shown on the Q-Q plot. (PDF) [file pgen.1006604.s002.pdf]

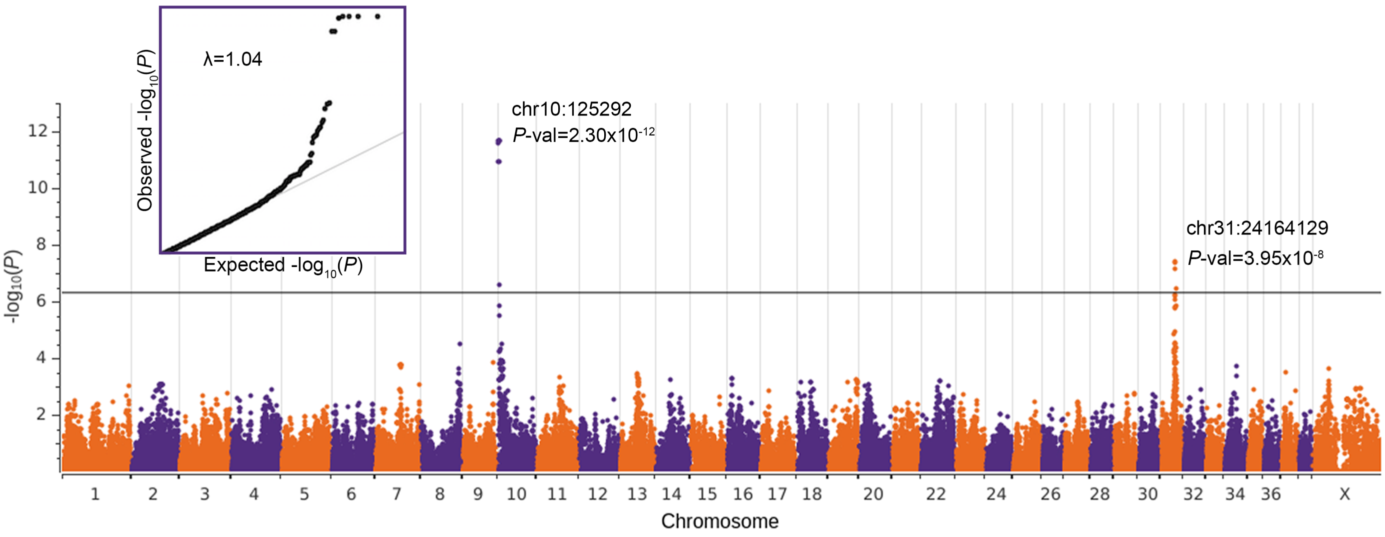

S2 Fig. Manhattan and quantile-quantile plots of combined DMS GWAS (97 cases vs. 68 controls). The –log10*P*-values (y-axis) for 98,520 Illumina SNPs are plotted against chromosome position (x-axis). The threshold for Bonferroni significance is shown as a black horizontal line. The *P*-value and position (canFam3) of the lead SNPs are reported. The Q-Q plot is boxed in purple and plots observed vs. expected Fisher's exact –log10*P*-values. The inflation factor (λ) is shown on the Q-Q plot.
