# Supplementary material for: Beyond the MHC: A canine model of dermatomyositis shows a complex pattern of genetic risk involving novel loci
Source: PLoS Genet. 2017 Feb 3;13(2):e1006604. doi: 10.1371/journal.pgen.1006604 (PMC5315411; doi:10.1371/journal.pgen.1006604)
Supplement: S3 Fig — UCSC 100 Vertebrates track for human chr21:29,130,846–29,130,860 showing the G insertion and seven base pair deletion created by MAP3K7CL indel (Dog c.383_392ACTCCACAAA>GACT). Bases in gray differ from the dog reference sequence. The canine sequence is highlighted in yellow. The RUNX3 binding motif is underlined. (PDF) [file pgen.1006604.s003.pdf]

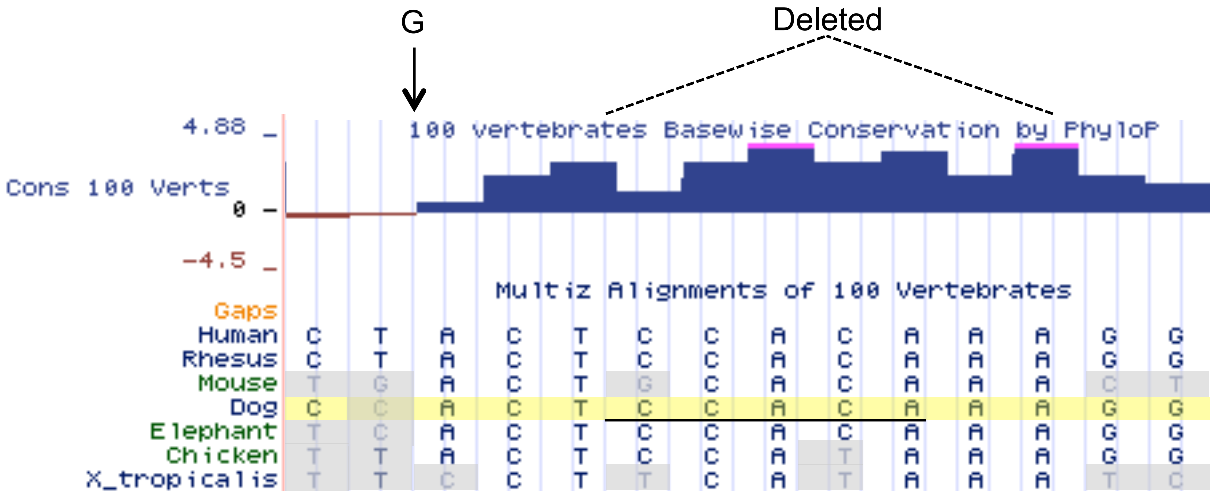

S3 Fig. *MAP3K7CL* indel with conservation and RUNX3 binding motif. UCSC 100 Vertebrates track for human chr21:29,130,846-29,130,860 showing the G insertion and seven base pair deletion created by *MAP3K7CL* indel (Dog c.383_392ACTCCACAAA>GACT). Bases in gray differ from the dog reference sequence. The canine sequence is highlighted in yellow. The RUNX3 binding motif is underlined.
