# Supplementary material for: Beyond the MHC: A canine model of dermatomyositis shows a complex pattern of genetic risk involving novel loci
Source: PLoS Genet. 2017 Feb 3;13(2):e1006604. doi: 10.1371/journal.pgen.1006604 (PMC5315411; doi:10.1371/journal.pgen.1006604)
Supplement: S4 Fig — Probability of disease (y-axis) for all combinations of PAN2 (x-axis) and MAP3K7CL genotypes are plotted in dogs (73 affected and 145 unaffected males; 59 affected and 245 unaffected females) (top) homozygous and (bottom) heterozygous for DLA- DRB1*002:01. (PDF) [file pgen.1006604.s004.pdf]

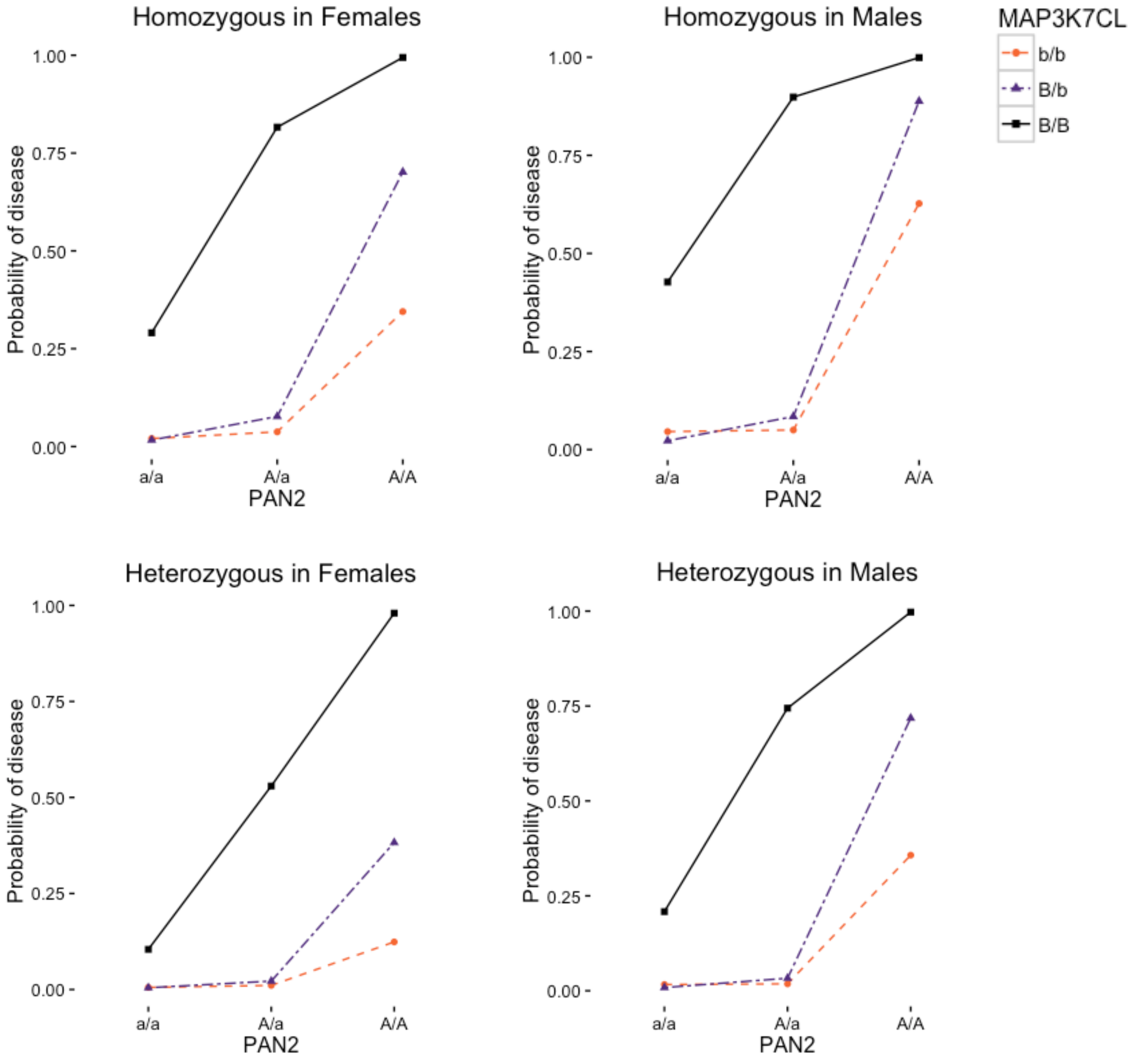

S4 Fig. Three-locus genotype disease probabilities by sex. Probability of disease (y-axis) for all combinations of *PAN2* (x-axis) and *MAP3K7CL* genotypes are plotted in dogs (73 affected and 145 unaffected males; 59 affected and 245 unaffected females) (top) homozygous and (bottom) heterozygous for *DLA- DRB1*002:01*.
