# Supplementary material for: Beyond the MHC: A canine model of dermatomyositis shows a complex pattern of genetic risk involving novel loci
Source: PLoS Genet. 2017 Feb 3;13(2):e1006604. doi: 10.1371/journal.pgen.1006604 (PMC5315411; doi:10.1371/journal.pgen.1006604)
Supplement: S5 Fig — Median age of onset is plotted for genotypes consisting of 2 (AAbb, aaBB, AaBb), 3 (AABb, AaBB), and 4 (AABB) risk alleles at PAN2 and MAP3K7CL. CC and Cc genotypes were combined for analyses. Number of individuals is shown to right. (PDF) [file pgen.1006604.s005.pdf]

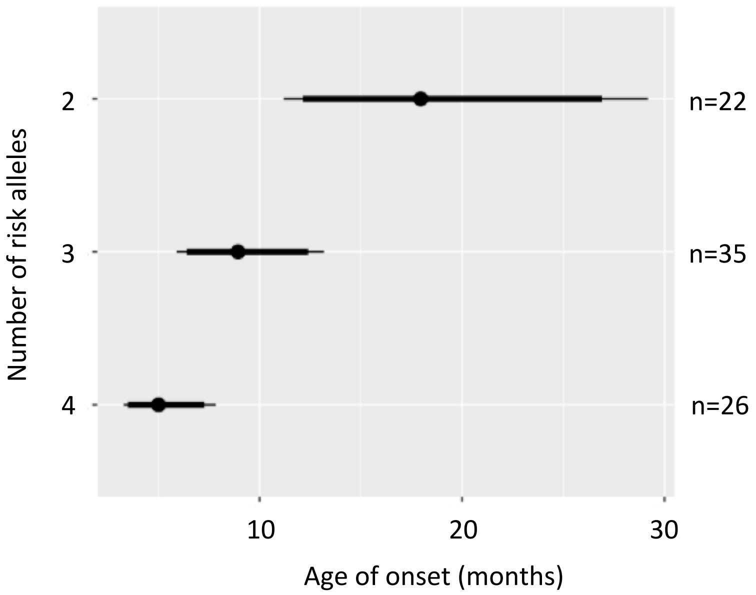

S5 Fig. Median age of onset for combinations of *PAN2* and *MAP3K7CL* genotypes. Median age of onset is plotted for genotypes consisting of 2 (AAbb, aaBB, AaBb), 3 (AABb, AaBB), and 4 (AABB) risk alleles at *PAN2* and *MAP3K7CL*. CC and Cc genotypes were combined for analyses. Number of individuals is shown to right.
