# Supplementary material for: Beyond the MHC: A canine model of dermatomyositis shows a complex pattern of genetic risk involving novel loci
Source: PLoS Genet. 2017 Feb 3;13(2):e1006604. doi: 10.1371/journal.pgen.1006604 (PMC5315411; doi:10.1371/journal.pgen.1006604)
Supplement: S6 Fig — ZH(p) values for all creeping windows containing 50 or more SNPs are plotted against chromosome position. Creeping windows are ≤1 Mb. (PDF) [file pgen.1006604.s006.pdf]

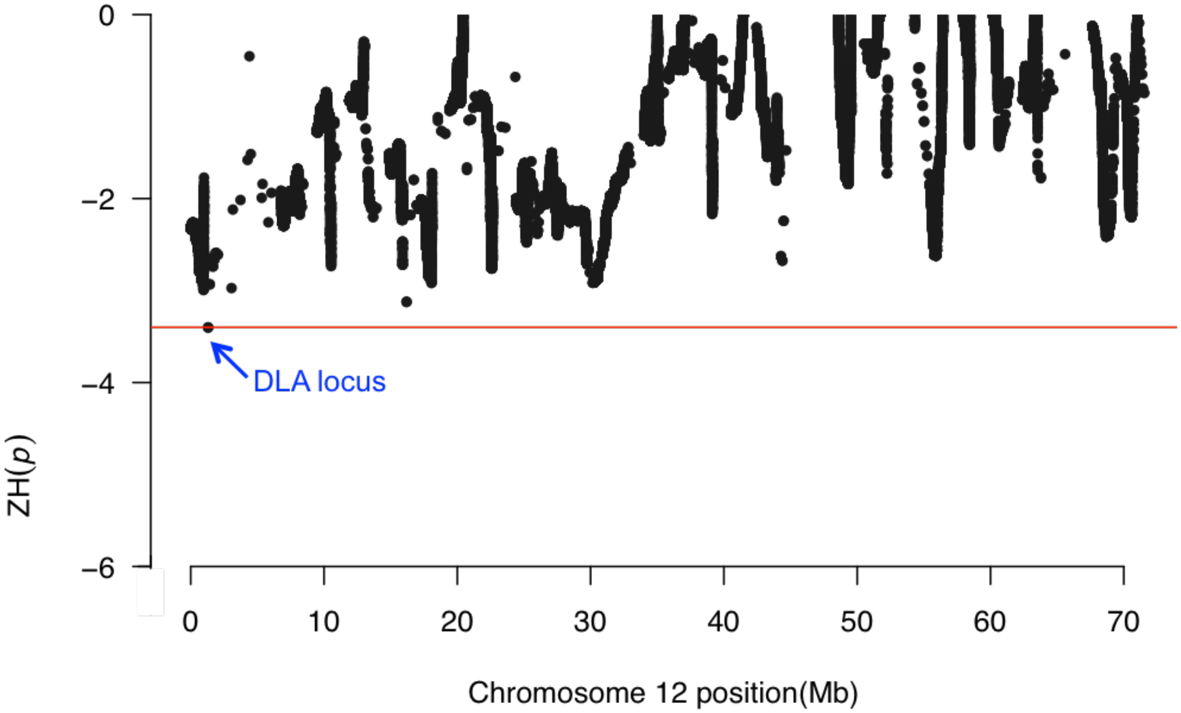

S6 Fig. Collie selective sweeps on chromosome 12. ZH(*p*) values for all creeping windows containing 50 or more SNPs are plotted against chromosome position. Creeping windows are ≤1 Mb.
