# Supplementary material for: Beyond the MHC: A canine model of dermatomyositis shows a complex pattern of genetic risk involving novel loci
Source: PLoS Genet. 2017 Feb 3;13(2):e1006604. doi: 10.1371/journal.pgen.1006604 (PMC5315411; doi:10.1371/journal.pgen.1006604)
Supplement: S1 Table — (PDF) [file pgen.1006604.s007.pdf]

S1 Table. SNPs exceeding Bonferroni significance (5.08X10-7).

COLLIE GWAS			SHETLAND SHEEPDOG GWAS			COMBINED GWAS		
CHR	BASE PAIR	*P*-VALUE	CHR	BASE PAIR	*P*-VALUE	CHR	BASE PAIR	*P*-VALUE
10	2163303	$1.47X10^{-8}$	31	24107856	$1.83X10^{-9}$	10	125292	$2.30X10^{-12}$
10	2125046	$4.24X10^{-8}$	31	24225299	$5.19X10^{-9}$	10	319019	$2.30X10^{-12}$
10	2170946	$4.24X10^{-8}$	31	24164129	$2.52X10^{-8}$	10	355241	$2.30X10^{-12}$
10	2453907	$4.24X10^{-8}$	31	23978661	$2.63X10^{-8}$	10	368251	$2.30X10^{-12}$
10	3757306	$4.24X10^{-8}$	31	24026411	$8.63X10^{-8}$	10	100589	$2.77X10^{-12}$
10	4413901	$4.24X10^{-8}$	31	24577081	$2.01X10^{-7}$	10	157505	$1.25X10^{-11}$
10	4460808	$4.24X10^{-8}$	31	24581581	$2.01X10^{-7}$	10	359293	$1.27X10^{-11}$
10	4482172	$4.24X10^{-8}$	31	24584079	$2.01X10^{-7}$	31	24164129	$3.95X10^{-8}$
10	1333693	$2.20X10^{-7}$	31	23847695	$2.20X10^{-7}$	31	24026411	$4.41X10^{-8}$
10	125292	$2.71X10^{-7}$	10	1720538	$2.56X10^{-7}$			
10	319019	$2.71X10^{-7}$	31	22033805	$3.33X10^{-7}$			
10	355241	$2.71X10^{-7}$	31	23925418	$3.88X10^{-7}$			
10	368251	$2.71X10^{-7}$						
10	2908436	$2.71X10^{-7}$						
10	4038486	$2.71X10^{-7}$						
10	4054430	$2.71X10^{-7}$						
10	4047342	$3.21X10^{-7}$						
