# Supplementary material for: Beyond the MHC: A canine model of dermatomyositis shows a complex pattern of genetic risk involving novel loci
Source: PLoS Genet. 2017 Feb 3;13(2):e1006604. doi: 10.1371/journal.pgen.1006604 (PMC5315411; doi:10.1371/journal.pgen.1006604)
Supplement: S2 Table — (PDF) [file pgen.1006604.s008.pdf]

S2 Table. Chromosome 10 variants segregating with the lead SNPs in the affected dogs.

Position	Reference	Alternate
983	C	T
1455	C	G
4302	A	G
4668	T	C
4860	A	G
4940	GATTCTTTTCAATTCGATTC	-
5145	C	T
5194	A	G
19674	A	G
19704	G	C
19913	C	T
20162	G	T
20220	T	G
20727	A	C
21565	T	A
24602	G	A
25645	G	A
25646	T	A
25650	G	A
28481	G	A
29441	C	T
29593	C	T
29747	A	G
30885	G	A
31306	G	A
31669	-	G
31689	A	C
31838	T	G
32200	G	C
33320	A	G
33783	-	TGTGTGTG
34721	G	A
35524	AAATAAAT	-,AAAT
35632	-	GATT
35919	A	G
35948	A	T
36416	G	A
36580	T	C
38490	A	G
38994	G	A
39186	T	G
39583	T	G
40824	-	G
41681	A	G
42722	-	TTTCTTTCTTTC
46324	G	A
46566	-	ACAT
47636	T	C
48860	G	A
51938	G	A
52373	A	T
53042	A	G
53231	A	T

53630	A	G
53669	A	T
54586	T	A
55154	C	G
55497	C	G
58074	A	G
58163	A	C
58193	C	T
58464	A	G
58750	C	G
58838	-	CC
61325	A	C
61350	C	G
62250	-	AAAT
62307	G	T
62348	C	A
62513	C	T
62583	A	G
65849	TTATTATATCCATTTCTTTTTTTTTTTTTTTTTATTA	-
67255	T	C
69315	G	T
70504	A	G
71227	-	A
71367	A	-
71494	G	A
72187	C	G
72799	T	G
73717	C	T
74999	G	A
75146	C	T
76869	A	C
77666	C	T
77841	C	T
77870	-	A
80439	-	GA
80594	T	A
81349	C	T
81424	T	G
82860	T	C
83789	T	C,G
84785	-	AA,AAATAA
84996	A	G
85168	A	-
86938	T	G
87675	CTCTCTCT	-,CTCTCTCTCTCT
87983	T	C
89056	T	G
89486	C	T
90648	-	A
91287	T	C
91288	G	C
92589	T	C
92627	C	T
95042	T	C
95875	A	G
95901	C	T

96173	-	TGTGTG
96654	-	T
97689	C	T
97690	C	T
100589	G	A
103427	T	G
105473	T	C
106427	GT	-
106429	-	ACACACACAC
108574	C	G
108782	A	G
109616	A	G
109776	G	C
114443	T	C
115789	T	C
115831	T	C
116599	A	G
117970	T	C
118024	T	C
119310	A	C
119614	T	C
121835	G	A
121966	-	G
122074	A	G
123135	TTT	-,T
123155	-	T
123784	C	T
124773	G	C
124785	G	T
125199	T	C
125292	A	G
125609	C	T
126124	A	T
126271	T	C
127268	-	C
127461	T	C
128048	G	A
130085	-	C
130214	C	A
130972	C	T
131270	A	G
132736	G	A
133034	-	CT
133275	G	A
133484	C	T
134896	TAAAT	-
135031	G	T
136172	A	G
137970	C	A
138038	T	A
138609	T	C
138649	G	A
138764	A	G
141322	-	C
141332	C	A
143362	-	A

144474	T	A
144542	T	G
146163	G	-
147307	TTTC	-
154392	C	T
155755	A	G
155779	C	A
155975	-	T
157505	A	G
158846	T	C
159056	C	-
159624	C	G
162915	-	AAA
163496	-	AA
163852	G	A
163991	C	T
164263	T	-
164266	-	ATCC
164267	TCC	-
164272	C	T
164273	C	T
164533	A	T
164534	A	C
164794	T	C
165665	C	A
165898	C	T
167713	A	-
168931	-	TAAA
168934	G	A
171502	T	G
171667	G	T
171742	C	T
172944	T	C
173571	-	AAC
174218	A	G
174553	-	A
175817	C	T
177122	A	G
178947	A	C
182833	G	T
183310	CT	-
187227	-	AA
188595	A	-
189225	-	CT
189269	T	A
189284	CC	-
189286	A	T
189791	T	A
190251	T	C
190571	TAAGAG	-
190578	TTATATCAGGGGATCCCTGGGTGGCGCAGCGGT TTGGTGCCTGCCTTTGGCCCAGGGCGCGATCC TGGAGACCCGGGATCGAATCCC	-
191464	T	C
191530	-	TT
191590	TT	-

192779	-	CC,CCCCC
192804	C	T
192869	A	-
193366	AAAAGTTTCTAAATGCTAAAAAAAAAAAAAAAAAA AAAAAAAAAAAAAAAA	-
194353	C	A
196673	-	GAGA
197178	C	G
197187	T	C
197579	TT	-
198004	CTTTTCTTTTCTTTTTTCTTT	-
198064	G	A
198073	C	T
198367	T	C
198717	A	C
198929	-	CTCTCTCTCTCTCTCTCT
199840	T	C
199868	C	T
203558	G	A
204436	T	C
205671	G	A
206024	C	T
206156	T	-
208223	G	A
209994	A	G
210826	A	-
211260	A	T
213933	G	T
219112	C	T
222809	T	G
223827	A	G
223963	T	C
227962	T	A
228189	T	C
230921	T	-
231747	C	G
232975	TAAAAATTT	-
233908	T	C
236429	T	C
236812	-	A
238792	AAG	-
238925	A	T
238934	C	T
239258	C	T
239760	G	A
240699	T	A
240737	-	CAGAGAGG
242049	G	A
244444	CTCT	-
245248	A	-
248834	G	T
249441	AAATAA	-
249645	G	C
251126	TG	-
251348	-	AAAT,AAATAAAT
251368	-	AATA

255125	A	C
255884	T	C
256328	G	C
256801	-	A
258954	-	A
258968	A	G
260700	-	TCTCTC
260805	G	A
263272	A	C
264609	A	-
264826	T	C
264891	CTCT	-
264966	-	A,AA
265620	-	ACATATGCT
265976	C	T
267300	-	GGCTAC
268234	G	C
272766	-	T
273866	A	-
277244	G	A
277609	T	C
279483	T	C
279819	-	T
283554	G	T,GT
284398	CT	-
288586	G	A
289277	C	A
289502	C	T
290259	C	T
292084	G	T
293323	T	G
293324	C	A
294584	A	G
297298	-	TTTTGT
297302	-	G
297305	-	TG
302341	-	ACACAC,ACACACACAC
304059	-	ATTT,ATTTATTT
309651	G	C
310721	G	C
313460	T	G
314549	A	-
315259	T	G
315772	G	A
316103	C	G
316273	C	G
317274	TTTA	-,TTTATTTATTTATTTATTTA
317694	T	C
319019	G	A
319508	A	G
319770	CC	-
319770	C	G
319771	C	A
321002	C	T
326105	G	A
327065	T	C

328251	T	G
328758	T	C
331135	A	G
331768	C	T
334964	-	TCTC
335222	-	A
337919	T	-
338118	-	T
338323	-	T
339097	C	T
342843	G	A
344853	A	G
344919	C	G
345210	-	CA
345919	A	G
346090	C	G
350369	-	AAAATAAAAT,AAAATAAAATAAAATAAAAT
351679	-	A
352840	A	G
355241	T	G
355949	G	T
355953	G	T
355969	T	G
355973	T	G
356507	C	A
358754	A	G
359293	A	G
363034	T	C
366583	A	G
368251	T	C
369609	T	C
370205	A	T
370561	-	AGAG,AGAGAGAGAG
370976	-	AA
371592	G	A
371755	C	T
372114	AA	-
376253	A	-
376823	-	T
376826	T	C
378943	G	T
380460	-	TATTC
381048	-	CT
382635	A	C
383033	-	T
384401	G	A
384806	T	C
385406	G	C
386021	A	T
387325	AAA	-
388036	-	T
388893	A	T
390674	TTA	-
391084	-	A
391262	C	T
392287	C	G

393819	A	-
394188	C	T
395522	AGTA	-
397303	C	T
397443	-	A
397839	T	G
397865	C	T
399997	AA	-
400111	T	C
400234	T	A
401025	-	AA
401521	TTTCTTTCTTTCTTTC	delTTTCTTTC,TTTCTTTCTTTCTTTCTTTCTTTCTTTCTTTC,TTTCTTTCTTTCTTTCTTTCTTTCTTTCTTTCTTTCTTTC
401970	A	C
403085	-	T
403733	A	T
407714	GA	-
407821	T	G
408176	G	C
408277	C	T
409137	-	CA
410451	G	C
411186	T	-
411648	A	C
425456	T	A
426511	T	G
435789	G	A
446629	G	A
450399	G	A
463902	T	A
464745	CA	-
468814	-	TTTC
473244	GT	-
479004	G	A
480891	-	AAAAAAAAAAAAAAAAAAAAAAAAAAAAAAAAAAAA
527236	A	-,AAA
529504	A	T
531522	G	A
539979	A	-
541546	G	T
545297	G	A
553356	C	-
559530	AGA	-
565958	G	C
569295	G	A
572024	G	A
584808	-	A
592283	A	-
592859	G	A
592863	T	A
596255	A	C
600296	ATTTTT	-
603725	-	A
606457	A	G
607719	-	ATTCTTTTTT

614684	G	C
621366	G	A
627760	G	A
628057	T	C
644479	C	T
679901	A	-
681353	T	A
684541	AAAGAAAAAGAAAAAGAA	-
685467	-	G
691560	AG	-
705625	ACAC	-,ACACACAC
708173	C	T
709584	T	A
713468	-	A
718727	A	G
721159	A	G
737228	G	-
746264	A	-
753324	G	A
754312	AG	-
754369	C	T
754507	-	ATTTATTT
759008	A	T
766773	C	T
770370	TTTATTTA	-
773874	-	T
773944	AC	-
774278	G	A
774287	-	TA
781273	TAAATAAA	-
791538	ATTT	-
792750	T	-
795887	T	C
800064	G	-
800906	TTGTT	-
801063	-	GA
808263	A	G
810666	C	T
811286	-	T
812076	CT	-,CTCTCT
812789	T	C
816416	A	T
834949	G	A
835850	T	C
838641	A	-
850906	CC	-,C
851959	C	-
855683	T	A
856040	C	T
863353	G	A
867832	G	A
871478	A	-
879196	AATAAATAAATA	-,AATA
883486	C	T
888939	T	-
890576	-	AATGAATAAATAAATAAATA

891964	A	-
893954	T	G
896459	-	AA
906950	-	A
910309	T	-
910992	T	C
917310	-	T
917884	T	A
919616	TTTTAT	-
923362	AAAT	-
926622	T	-
927308	A	G
929039	-	TTAT
933041	TT	-
940501	-	A
940996	A	G
941544	G	A
949170	A	G
962343	TATTTATT	-
964052	G	A
965072	-	TTTA
965128	AGAG	-
971007	TGTTT	-
971895	T	-
974559	A	T
975201	T	C
975203	C	T
975216	C	A
978940	C	T
993657	T	C
1010309	-	CT
1020038	TTC	-
1024911	-	T
1025010	-	AGAG
1031837	C	T
1042098	TATG	-
1051909	T	C
1061145	C	-
1074035	T	C
1090400	-	TTTA,TTTATTTA
1096770	G	A
1096919	A	T,AT
1099689	ATAA	-
1108799	C	T
1111745	-	ATTT
1121749	G	A
1127082	C	T
1128658	-	T
1130331	-	AC
1131360	C	T
1131903	-	C
1135206	A	G
1150305	T	C
1164726	C	T
1165045	-	AG
1168367	T	C

1175038	T	C
1182874	TCTC	-
1195393	-	TC
1207836	-	A
1217285	-	CA
1222163	T	-
1228277	G	C
1239562	G	A
1259335	T	A
1272949	G	A
1286150	C	T
1290104	C	T
1291016	C	T
1296029	G	A
1299356	C	T
1315005	C	A
1319925	G	C
1322218	G	A
1325422	G	A
1333693	G	A
