# Supplementary material for: Beyond the MHC: A canine model of dermatomyositis shows a complex pattern of genetic risk involving novel loci
Source: PLoS Genet. 2017 Feb 3;13(2):e1006604. doi: 10.1371/journal.pgen.1006604 (PMC5315411; doi:10.1371/journal.pgen.1006604)
Supplement: S3 Table — (PDF) [file pgen.1006604.s009.pdf]

S3 Table. Chromosome 31 variants segregating with the lead SNPs in the affected Shetland sheepdog.

Position	Reference	Alternate
24026411	T	C
24026652	A	C
24026717	-	TAAGAGGAG
24026740	T	C
24026756	G	A
24026797	A	T
24026835	G	A
24026878	C	T
24026886	C	T
24027079	G	T
24027126	T	C
24027219	G	A
24027267	G	A
24027324	T	C
24027430	G	A
24027565	AACT	-
24027640	C	T
24027679	-	CAA
24027791	G	T
24027957	G	T
24028241	A	G
24028305	G	-
24028334	G	A
24028339	C	A
24028560	-	AAA
24028649	-	GA
24028659	T	C
24028900	A	G
24028918	C	A
24029003	C	T
24029289	C	T
24029465	A	C
24029536	G	T
24029568	T	-
24029571	-	ATAAATAAATAAATAAAT
24029606	AA	-
24029785	T	C
24029985	C	T
24030534	C	T
24030579	C	-
24030609	T	A
24030637	C	G

24030831	C	G
24031216	C	G
24031256	G	C
24031323	T	C
24031395	TCGT	-
24031416	G	A
24031438	G	A
24031603	T	C
24031706	T	G
24031752	ATC	-
24032222	T	C
24032272	G	A
24032388	AT	-
24032416	G	C
24032454	T	C
24032683	-	TAGTT
24032727	G	A
24032746	A	G
24032757	G	A
24032933	T	A
24033056	C	T
24033162	C	G
24033221	C	G
24033274	C	G
24033279	T	C
24033290	GTGG	-
24033292	GG	-
24033317	-	AT
24033321	TT	-,TTT,T
24033417	AAG	-
24033460	-	AAA
24033501	-	AAA
24033638	C	T
24033683	G	T
24033707	T	C
24033758	-	AGA
24034032	G	C
24034045	G	T
24034053	AT	-
24034069	T	A
24034145	A	G
24034151	G	A,C
24034251	AAA	-,A
24034309	C	A
24034313	T	G

24034449	G	T
24034450	C	T
24034930	A	C
24035008	TTTT	-
24035493	T	-
24036030	T	G
24036039	T	G
24036350	C	T
24036378	G	A
24036460	-	TGTTTTT
24036501	A	C
24037799	T	C
24038043	C	G
24038172	T	A
24038259	T	-
24038599	T	C
24039260	TATC	-
24039311	G	A
24039958	G	A
24040074	T	C
24040093	A	G
24040611	C	T
24041158	T	C
24041225	A	C
24041230	T	G
24041665	A	G
24041670	T	A
24041726	A	G
24043229	G	A
24044608	G	C
24044728	T	C
24044957	G	T
24045781	-	T
24045782	-	CAGAGAGAT
24047545	T	G
24047547	T	A
24047768	G	A
24049153	G	A
24049689	ATTT	-
24049813	-	T
24050897	A	C
24051358	A	G
24051368	G	T
24051597	G	A
24052413	T	G

24056097	TTTTTT	-
24056253	G	A
24056422	C	A
24057038	G	A
24057374	A	G
24058424	A	G
24058660	G	C
24059179	C	A
24059596	G	C
24059877	G	C
24060268	-	A
24060716	A	-
24060891	-	GAATTCCCAG
24060893	TCT	-
24061063	G	C
24061204	AT	-
24061543	T	C
24061866	A	T
24062054	G	-
24062306	T	C
24062312	-	T
24062559	T	A
24063136	G	A
24063722	G	C
24063880	C	A
24063942	-	T
24063949	G	T
24064116	TGCCTCTGTG	-
24066188	T	A
24066346	-	T
24066648	A	T
24066660	A	T
24066664	A	T
24066668	A	T
24066744	T	G
24067158	T	G
24067185	T	C
24067220	C	T
24067256	C	T
24068039	T	C
24069628	G	A
24069657	C	T
24070825	C	T
24070938	C	T
24071381	C	T

24071511	-	AA
24071562	-	AAC
24071822	A	G
24071827	G	A
24072388	ATCCATAAAGG	-
24072480	CTGT	-
24072557	G	A
24072929	T	C
24073144	AAC	-
24073205	A	T
24073241	AA	-
24073399	C	G
24073466	A	T
24073467	A	C
24073515	G	A
24074150	C	A
24074221	-	ACAG
24074277	T	G
24074333	GT	-
24074337	-	AAACCGCC
24074516	G	T
24074572	C	G
24074581	G	A
24074583	ACA	-
24075314	TG	-
24075316	-	T
24075318	T	G
24075450	G	T
24075530	G	A
24075597	C	T
24075625	TTT	-
24075647	G	A
24075676	G	A
24075938	-	TT
24075940	-	C
24076011	TTTTTTTTTA	-
24076019	TA	-
24076025	C	A
24076184	-	T
24076200	-	GGG
24076203	T	-
24076208	TTTC	-
24076270	A	T
24076400	-	A,ATTTA
24076403	-	A

24076403	T	A
24076405	-	TA,TATTTA
24076407	T	A
24076430	-	AGAGAG
24076456	G	C
24076475	GGAGAA	-
24076492	G	A
24076533	G	-
24076592	A	G
24076779	G	A
24076802	C	T
24076919	G	A
24076921	C	T
24077000	AACCCAAAAATAAAAAAA	-
24077010	T	A
24077017	A	T
24077031	A	G
24077547	C	A
24077564	-	TTT
24077574	A	T
24077591	A	G
24077768	-	ACAG
24077896	A	G
24077906	CCTCTG	-
24077945	-	T
24077963	A	T
24077991	G	C
24078090	A	C
24078173	G	C
24078235	A	G
24078240	A	G
24078282	G	A
24078306	C	T
24078308	G	C
24078329	G	A
24078392	G	T
24078407	G	T
24078481	A	T
24078492	C	G
24078532	T	-
24078576	C	T
24078655	C	A
24078677	T	C
24078861	C	-
24079073	TC	-

24079097	T	C
24079495	A	C
24079559	G	A
24079596	-	T
24079621	C	-
24079624	AACCTAACCTAAC	-
24079648	-	T
24079804	T	C
24079814	T	C
24079915	A	G
24080158	A	G
24080440	G	C
24080888	A	G
24081108	-	A
24081134	-	A
24081396	A	G
24081431	C	T
24081480	A	G
24081552	T	G
24081579	C	A
24081665	C	T
24081672	T	C
24081825	A	G
24081953	C	T
24081977	A	G
24082006	-	GAAGGATGACGGC
24082062	T	C
24082091	G	A
24082173	C	T
24082214	C	T
24082229	C	T
24082232	G	A
24082245	C	T
24082273	C	T
24082507	A	C
24082572	T	A
24082785	C	T
24082900	-	G
24083036	C	T
24083129	T	G
24083357	T	C
24083548	T	C
24083627	C	G
24083730	C	T
24083747	C	T

24083870	A	G
24084159	G	A
24084447	T	C
24084574	-	T
24084576	A	T
24084619	G	A
24084765	T	A
24084863	C	T
24084927	C	T
24084940	-	A
24084993	T	G
24085048	T	C
24085055	A	G
24085124	T	C
24085156	A	G
24085164	A	G
24085216	T	C
24085386	T	C
24085722	G	A
24085963	G	A
24086038	T	C
24086043	T	A
24086075	A	C
24086203	A	G
24086378	A	T
24086387	G	A
24086462	T	G
24086671	A	C
24086982	TTTT	-
24087052	T	G
24087096	AT	-
24087149	AGG	-
24087178	-	T
24087233	A	G
24087528	T	C
24087811	G	A
24088316	T	A
24088383	G	A
24088561	G	T
24088777	G	C
24088821	G	A
24089062	G	A
24089097	C	T
24089155	G	C
24089984	C	A

24089997	-	T
24090031	ATTT	-
24090035	-	ATTTATATTTTAAAG
24090041	-	A
24090043	-	TTAAAG
24090237	G	A
24090789	-	A,AA
24091436	-	G
24091688	T	A
24092344	-	GA
24092351	-	TG
24092428	A	G
24092732	G	A
24093142	G	A
24093223	G	A
24093251	T	-
24093349	G	T
24093424	C	T
24093426	C	T
24093715	C	T
24093762	C	T
24093926	A	T
24093989	G	A
24094447	A	G
24094466	A	T
24094540	G	A
24094602	G	A
24094652	TAAAAATTGTTAAAAAAAAAAAAAAAAGACAAATAAA AAAAAAAAAA	-
24095018	T	C
24095063	T	A
24095070	C	T
24095077	T	C
24095212	G	T
24095508	G	A
24095511	C	T
24095709	A	G
24095945	A	G
24096017	A	G
24096138	T	A
24096214	A	G
24096257	C	T
24096305	T	C
24096382	T	C
24096401	G	A

24096447	AAA	-,GAAAAA
24096553	T	C
24097012	C	T
24097064	T	C
24097221	T	C
24097259	G	T
24097264	TTT	-,TT
24097303	A	G
24097402	G	A
24097418	G	A
24097494	G	T
24097734	-	A
24097737	C	T
24097747	G	A
24097871	G	T
24097885	T	C
24097887	C	T
24098041	G	A
24098283	TC	-
24098335	G	A
24098354	A	T
24098386	G	T
24098440	T	A
24098520	T	C
24098551	T	C
24098568	T	C
24098689	A	G
24098780	G	A
24098933	A	G
24099098	T	C
24099101	G	A
24099116	G	A
24099148	T	C
24099164	A	T
24099167	CT	-,CTCTCTCTCT
24099217	A	-
24099237	G	T
24099254	G	A
24099412	C	T
24099613	C	T
24099670	G	A
24099714	TATA	-
24099724	T	A
24099748	G	A
24099781	A	G

24099807	C	T
24099874	G	A
24099936	G	A
24099979	G	T
24099998	T	A
24100160	T	A
24100208	A	G
24100340	C	T
24100460	G	T
24100546	C	T
24100586	T	C
24100596	T	C
24100754	G	A
24100761	A	G
24100795	T	C
24100937	G	A
24100970	C	T
24101009	C	T
24101926	A	-
24102001	-	C
24102118	T	C
24102422	T	G
24102425	A	G
24102788	T	G
24102849	AAAG	-,AAAGAAAG,AAAAGAAAG
24102995	A	G
24103908	G	A
24103961	A	T
24104043	C	T
24104261	TTT	-
24104554	A	T
24104555	T	A
24104573	T	A
24104786	A	-
24104788	T	C
24104790	C	G
24104796	A	G
24104797	G	C
24104801	-	A
24104805	T	G
24104856	G	T
24104914	C	A
24104963	G	A
24104965	A	T
24105818	AGAG	-

24105831	AGAGGG	-
24105844	G	A
24105845	A	C
24106166	AT	-
24106175	T	G
24106241	G	A
24106299	C	T
24106445	T	C
24106933	G	T
24107050	T	C
24107150	C	T
24107190	C	T
24107215	TA	-
24107230	A	C
24107231	G	A
24107233	A	-
24107259	C	T
24107332	T	C
24107351	C	T
24107489	C	T
24107528	C	T
24107554	T	C
24107686	G	A
24107856	A	G
24107873	T	A
24107991	T	C
24108130	T	C
24108229	C	A
24108236	A	G
24108256	C	T
24108276	-	CCC
24108301	A	G
24108370	C	T
24108417	GGATTAA	-
24109139	A	G
24109315	-	ACAC
24109562	C	T
24109614	A	T
24110152	G	T
24110251	G	T
24110252	A	G
24110277	A	G
24110350	T	C
24110466	C	T
24110506	T	C

24110594	A	G
24110606	C	T
24110652	C	A
24110685	G	A
24110734	A	T
24110979	G	C
24111007	G	T
24111017	C	T
24111018	G	A
24111020	G	A
24111052	G	T
24111536	G	A
24111620	A	C
24111758	G	A
24112075	TCTGGTGTGCTAAGC	-
24112187	A	G
24112191	C	A
24112287	A	G
24112306	T	C
24112390	C	T
24112612	A	T
24112686	C	T
24112748	A	C
24112818	T	C
24112848	A	G
24112860	T	A
24112861	T	G
24112930	A	G
24113013	G	A
24113027	C	T
24113067	TT	-,T
24113164	G	A
24113283	C	A
24113284	C	T
24113324	T	C
24113331	CCGC	-
24113335	C	T
24113342	C	A
24113411	TCTCTCTC	-,TC
24113421	T	-
24113423	TCTCT	-
24113454	-	T
24113698	T	C
24113713	G	A
24113812	G	A

24113818	G	A
24113893	T	G
24113895	G	A
24114292	T	C
24114304	T	C
24114345	A	G
24114405	T	A
24114926	T	G
24114954	C	T
24115003	T	A
24115048	C	T
24115525	G	A
24115650	-	TTGT
24116098	A	G
24116168	A	T
24116208	A	G
24116264	A	-
24116382	T	-
24116395	C	A
24116422	-	TATT,TATTTATT
24116533	A	T
24116724	C	T
24116756	A	C
24116757	G	T
24116764	A	G
24116792	C	T
24116835	G	A
24116872	T	A
24117196	A	-
24117591	-	AG
24117676	C	T
24117718	C	T
24118374	A	G
24118829	A	G
24118919	A	G
24119144	T	C
24119184	C	A
24119352	T	-
24119398	G	A
24119866	A	G
24119882	G	A
24119908	G	A
24120054	TC	-
24120154	G	A
24120325	-	C

24120439	T	A
24120583	A	C
24120907	C	T
24121089	C	T
24121106	G	A
24121133	C	T
24121135	C	T
24121165	C	T
24121172	T	C
24121233	T	C
24121275	G	C
24121315	A	G
24121380	T	C
24121441	C	T
24121627	-	T
24121685	CG	-
24121689	CA	-
24121734	G	A
24122032	A	G
24122216	T	-
24122556	T	C
24122637	G	T
24122688	A	G
24122697	G	A
24122775	-	T
24122804	GA	-
24123464	A	T
24123775	C	A
24124086	T	-
24124237	T	G
24124252	T	A
24124477	A	T
24124530	TTTT	-
24124773	G	T
24124798	T	C
24124806	G	A
24124842	A	C
24124868	C	T
24124940	T	C
24124983	A	G
24126005	C	T
24126330	-	A
24126380	C	T
24126497	-	C
24126497	T	C

24126580	-	AAGAGCTTGGCTCTTAAA
24127292	T	G
24127419	GC	-
24127422	-	ACT
24127430	-	C
24127502	TT	-,T
24127555	A	G
24127599	C	G
24127791	G	A
24127963	G	T
24127995	G	A
24128148	T	G
24128409	ACC	-
24128568	-	TTA
24128678	A	G
24129467	G	A
24129627	A	G
24129649	T	C
24129720	T	C
24130406	C	T
24130412	T	A
24130433	G	A
24130483	A	T
24130961	T	C
24131274	C	T
24131280	T	C
24131492	T	C
24131493	C	T
24131647	TAAG	-
24131673	A	G
24131802	C	T
24132273	-	G
24132276	CCACAAA	-
24132343	A	C
24132422	G	C
24132546	A	G
24132560	T	C
24132802	-	T
24133211	-	TG
24133545	C	T
24134574	G	T
24134575	T	A
24134652	T	C
24135523	C	G
24136025	A	C

24136253	A	G
24136310	AAAAG	-
24136769	C	T
24136822	A	G
24136944	-	G
24137294	G	A
24137329	A	G
24137406	A	G
24137537	C	T
24137591	TT	-,T
24137875	C	G
24138645	A	G
24138739	A	G
24138745	-	G
24138766	A	G
24138791	-	C
24138795	G	-
24138796	G	A
24138846	-	T
24139325	TCTTTCTTTCTTTC	-
24139333	TCTTTC	-
24139337	TC	-
24139466	-	AA
24139866	TATT	-,TATTTATT
24139888	T	C
24140107	C	T
24140199	-	T
24140200	A	T
24140220	A	G
24141367	C	A
24141382	A	-
24141389	-	T
24141395	-	AAC
24141396	G	T
24141399	T	C
24141647	C	T
24141712	G	C
24141824	T	A
24141868	T	C
24142531	A	G
24143184	C	T
24143697	G	A
24143967	G	A
24143971	-	CG
24144962	-	G

24144967	-	G
24145189	C	T
24145190	G	A
24145236	C	G
24145513	A	G
24145549	ACTC	-
24146752	G	A
24146982	G	A
24147255	T	C
24147384	A	C
24147630	-	TTTA
24147742	G	A
24148218	C	T
24148412	GCTCT	-
24149112	T	C
24149254	T	C
24150701	G	A
24150726	G	C
24150744	A	C
24150798	C	T
24151077	-	CTG
24151203	T	C
24151321	TTT	-,T
24151940	G	A
24151970	G	A
24151971	T	C
24151973	G	-
24151983	A	G
24153094	C	G
24153129	-	C
24153258	T	A
24153300	-	T
24153389	C	T
24153392	A	C
24153436	C	T
24153452	C	T
24153653	T	C
24154382	C	G
24154507	C	A
24154548	T	-
24154743	T	G
24154912	A	-
24154966	G	C
24154976	C	A
24155315	G	A

24155688	C	A
24155995	A	T
24156162	C	T
24156202	C	G
24156310	-	G
24156465	A	G
24156508	A	G
24156583	T	C
24156829	T	C
24156834	A	T
24157035	C	T
24157044	T	C
24157065	T	C
24157102	G	T
24157103	A	T
24157405	C	T
24157510	A	C
24157516	C	T
24157764	-	T
24158278	GT	-
24158293	T	C
24158945	A	T
24159157	-	GAGAGAGAGAGAGAGAAA
24159432	A	G
24159476	G	A
24159761	C	T
24159929	G	A
24160075	-	AA
24160106	T	G
24160233	T	-
24160407	A	T
24160521	C	T
24160862	-	C
24160865	C	A
24160867	T	C
24160880	CCACTGGCCC	-
24160936	C	T
24161131	G	A
24161144	C	T
24161146	T	A
24161170	G	T
24161271	T	C
24161287	G	A
24161314	G	A
24161322	G	A

24161578	T	C
24161609	A	C
24161635	-	TT
24161753	A	G
24161771	G	A
24161816	A	T
24161989	G	A
24162035	G	A
24162047	G	A
24162082	T	G
24162090	G	A
24162179	TTGG	-
24162229	G	-
24162230	A	T
24162393	T	C
24162407	T	G
24162503	A	T
24162540	-	G
24162626	T	C
24162717	A	G
24162868	-	A
24162877	A	G
24162906	A	G
24162926	T	C
24162927	C	A
24162928	G	A
24163084	A	G
24163105	C	-
24163255	G	A
24163283	T	C
24163306	T	C
24163326	G	A
24163450	C	T
24163458	C	T
24163502	C	A
24163621	A	T
24163649	G	A
24163664	C	A
24163682	A	T
24164129	C	T
24164192	C	T
24164616	AAAAA	-,AAA,AAAA
24164633	G	A
24164655	T	G
24164659	-	G

24164785	G	A
24164834	C	G
24165070	T	C
24165370	-	TATT
24165638	C	A
24165967	G	A
24166000	G	A
24166051	C	T
24166084	G	A
24166220	C	A
24166264	C	G
24166419	G	A
24166428	G	A
24166446	T	A
24166556	G	A
24166558	A	G
24166575	T	C
24166654	T	C
24166679	A	G
24166751	C	A
24166771	A	G
24166788	G	A
24166797	G	A
24166978	T	C
24167019	G	A
24167488	C	T
24167697	C	A
24168088	-	GGGG
24168096	-	GGA
24168138	G	A
24168144	A	G
24168564	A	G
24168615	C	T
24168797	T	C
24168807	C	A
24168808	A	G
24168882	GTTTT	-
24168889	T	G
24168891	TTA	-
24168896	T	-
24168898	T	G
24169059	G	A
24169152	C	T
24169232	A	C
24169254	T	A

24169255	G	A
24169335	G	A
24169426	A	C
24169751	A	C
24169864	G	C
24169946	A	G
24170149	A	G
24170208	A	G
24170213	T	A
24170461	C	A
24170498	G	A
24170698	C	T
24170833	A	G
24170880	A	T
24170982	G	T
24171289	-	TTTTCTTTTCTTC
24171296	-	TTC
24171305	-	TTTCTTTTC
24171347	AGAG	-
24171510	C	T
24171512	C	G
24171554	A	T
24171632	C	T
24171700	G	A
24171797	A	G
24171936	C	T
24172160	C	T
24172389	T	G
24172574	C	T
24172716	-	CTAA
24173265	G	A
24173500	TTT	-
24173515	T	C
24173859	A	G
24173934	A	C
24174123	-	G
24174305	-	G
24174306	G	-
24174469	C	G
24174535	G	A
24174540	C	T
24174544	C	T
24174932	T	C
24174988	C	T
24175276	AAA	-

24175466	G	A
24175792	G	A
24176086	G	C,T
24176092	C	T
24176322	G	A
24176326	T	G
24176745	G	A
24177201	C	-
24177624	C	T
24177674	G	A
24177679	T	C
24177789	C	T
24178017	C	T
24178097	T	C
24178985	C	G
24179028	C	T
24179712	T	C
24179725	G	A
24179853	-	CAGA
24179903	C	T
24179906	T	C
24180366	T	C
24180379	T	C
24180701	C	T
24181836	T	-
24181838	GAGGA	-
24184044	G	A
24184058	C	T
24184569	C	T
24184626	A	-
24184645	G	C
24184686	G	A
24184927	C	G
24185101	G	A
24185128	C	T
24185129	A	G
24185202	G	A
24185377	G	-
24185423	G	T
24185424	A	G
24185554	A	G
24185654	A	G
24185767	TT	-,T
24185813	-	AG
24185941	C	G

24186065	T	C
24186135	C	T
24186143	C	T
24186381	-	CACT,CACTCT,CTCT
24186392	AAA	-
24186488	G	A
24186519	A	C
24186525	G	T
24186747	A	T
24186902	C	T
24186978	T	-
24187048	A	G
24187122	AGAC	-
24187286	G	T
24187290	T	A
24187565	C	T
24187658	T	A
24187909	A	C
24187928	A	G
24188273	G	A
24188549	T	A
24189711	T	C
24190074	G	A
24190500	-	A
24190682	G	A
24190881	C	-
24191331	T	C
24191416	C	A
24191461	T	C
24191568	T	C
24191755	-	CTCTCT
24191795	T	-
24191999	T	C
24192149	C	A
24192679	C	T
24193123	T	A
24193216	G	A
24193245	G	A
24193571	AG	-
24193574	G	A
24193750	C	T
24193998	T	C
24194053	A	G
24195687	G	A
24195805	-	TT

24195917	TT	-,T
24195932	TA	-
24196099	G	A
24196136	A	G
24196397	G	A
24196427	G	T
24196641	C	T
24196929	A	G
24197073	T	G
24197240	T	G
24197570	C	T
24197947	T	G
24197948	G	A
24198039	G	T
24198108	C	T
24198123	A	G
24198178	G	A
24198307	A	G
24198834	G	C
24198857	A	G
24199002	T	-
24199009	T	G
24199203	-	A
24199207	-	AC
24199211	CC	-
24199211	C	A
24199276	G	A
24199812	-	T
24200198	C	T
24200589	A	-
24200637	C	T
24200921	-	A
24201213	T	C
24201257	G	A
24201381	C	-
24201383	GTC	-
24201656	C	T
24201803	G	A
24201874	G	-
24201897	G	A
24202086	C	A
24202117	T	C
24202582	-	AGACATG
24202693	T	G
24202808	-	A

24203047	A	G
24203076	T	C
24203388	AA	-
24203402	A	T
24203574	C	T
24203704	T	C
24203994	G	A
24204356	C	A
24204551	A	G
24204919	G	A
24205112	GATA	-
24205366	AA	-,A
24205515	T	C
24205675	-	T
24205888	GTTTCTTG	-
24206115	G	A
24206155	G	A,T
24206290	T	G
24206966	T	G
24207275	T	G
24207359	G	A
24207362	T	C
24207515	A	T
24207584	A	G
24207700	-	TAT
24207993	A	T
24209114	C	T
24209171	G	-
24209182	G	T
24209341	C	T
24209414	G	T
24209446	A	T
24209508	T	A
24209691	A	G
24209712	G	A
24209768	-	GA,GAGA
24210279	-	A
24210348	A	G
24210439	CTAGA	-
24210785	A	G
24210947	C	A
24211178	C	G
24211330	A	G
24211400	A	G
24211769	T	-

24211840	T	-
24211849	G	T
24212013	T	C
24212046	-	T
24212122	C	G
24212214	A	G
24212568	G	T
24212570	G	A
24212788	T	C
24213227	T	C
24213282	T	-
24213542	A	G
24214444	G	A
24214586	-	C
24214596	A	T
24214631	T	C
24214692	A	G
24214693	C	T
24214841	T	-
24214844	-	A
24214851	-	ATTTTTTA
24214911	C	T
24214924	G	A
24215007	G	-
24215009	C	A
24215199	-	AATC
24215372	GC	-
24215372	G	A
24215375	-	TG
24215827	-	A
24216430	A	G
24216458	G	A
24216525	T	C
24217244	A	C
24217466	C	T
24217605	-	CTCT
24217624	A	G
24219165	C	T
24219558	-	C
24219621	A	G
24220280	T	C
24220713	T	-
24221687	T	A
24221875	G	A
24221946	G	A

24222135	G	T
24222173	TTTT	-
24222187	-	TA
24222189	TT	-
24222191	-	AAAAA
24222455	GAAA	delGAAAGAAAGAAAGAAA,GAAAGAAAGAAAGAAAGAAA
24222973	TTTC	-
24222990	A	-
24222992	CTATCTA	-
24223142	C	A
24223619	G	A
24224272	A	T
24224446	T	C
24224603	G	A
24224641	T	A
24224688	C	T
24224846	C	T
24224958	A	-
24225075	G	A
24225095	G	A
24225114	G	A
24225140	C	T
24225194	A	T
24225207	T	C
24225299	C	T
24225495	-	A
24225500	T	A
24225738	A	G
24225801	A	T
24226287	G	A
24226582	T	C
24226715	-	T
24226720	G	T
24226992	A	G
24227157	T	G
24227546	A	G
24227915	A	G
24227992	T	G
24228040	C	T
24228254	A	G
24228355	C	G
24228662	A	G
24228886	-	AA
24228901	C	-

24229045	C	T
24229141	C	T
24229260	G	A
24229336	G	A
24229506	C	T
24230060	A	-
24230312	A	G
24230349	T	C
24230406	T	C
24230459	-	AAGAAAGTTAGTGTAT
24230685	A	G
24230834	C	T
24231618	A	G
24231692	A	T
24231966	C	T
24232025	T	C
24232241	C	T
24232305	-	A
24232377	C	T
24232452	AG	-,AGAG
24232687	C	T
24232853	-	T
24232862	A	T
24233062	T	-
24233063	-	TTTATTTTATTTTATTTTATTTTATTTTATTTTA
24233292	G	A
24233337	C	T
24233494	-	T
24233562	G	A
24233591	G	A
24233884	TGTGTGTG	-,TG
24233955	GATA	-
24233999	A	G
24234098	-	TT
24234456	G	A
24234904	TT	-,TTTTTT
24235041	G	A
24235304	-	TCTC
24235467	C	T
24235570	C	T
24235717	TTTTTTTAATGTTTA	-
24235766	-	AG,AGAGAG
24235793	T	C
24235803	G	A
24235941	A	T

24235966	A	T
24236090	A	G
24236111	G	A
24236184	T	C
24236404	G	C
24236621	-	TATTTATT,TATTTATTTATT,TATTTATTTATTTATT
24236711	TTTTTTT	-
24236945	C	G
24237213	A	G
24237222	T	C
24237316	T	C
24237338	C	T
24237513	A	T
24237517	-	GGCAAT
24237634	A	G
24237811	TTTG	-
24238100	G	C
24238208	T	G
24238441	A	T
24238524	C	A
24238526	G	A
24238532	G	T
24238533	T	C
24238534	G	A
24238556	-	T
24238757	G	A
24239718	T	C
24239910	C	T
24240104	-	T
24240121	G	T
24240150	TT	-
24240162	G	T
24240165	G	T
24240224	C	T
24240352	T	G
24240358	T	C
24240893	T	C
24241068	A	G
24241404	C	A
24241806	G	A
24241833	T	C
24241912	C	T
24241992	T	A
24242025	T	C
24242056	C	T

24242060	A	G
24242147	T	C
24242219	T	G
24242314	C	T
24242335	A	T
24242438	C	T
24242527	T	C
24242716	A	T
24242895	C	T
24243030	A	T
24243057	A	C
24243538	A	-
24243582	G	A
24243719	C	T
24243748	G	A
24243857	T	C
24244133	G	T
24244233	AT	-
24244337	C	T
24244397	C	T
24244476	G	A
24244630	T	C
24244967	T	A
24245433	C	T
24245638	G	A
24245673	C	T
24246390	TT	-
24246462	T	A
24246937	G	A
24247120	T	-
24247122	C	A
24247290	-	CT
24247632	TGCTGCAAAAATAGGTACAGTATGTAAAAATGC	-
24247672	G	C
24247673	T	A
24248312	A	T
24248472	G	A
24248714	T	-
24248748	AAAAT	-
24248811	C	T
24248822	T	C
24248840	-	GA
24248917	A	G
24248934	A	T
24248938	C	T

24248985	C	T
24249056	A	G
24249094	C	T
24249106	A	G
24249161	A	G
24249287	C	T
24249295	T	A
24249343	A	G
24249633	C	T
24249860	T	C
24249870	G	A
24250006	G	-
24250158	T	A
24250187	AC	-
24250196	C	G
24250262	C	T
24250400	G	A
24250401	C	T
24250438	T	C
24250543	G	A
24250723	A	G
24250857	-	T
24250977	C	T
24251285	-	TGTT
24251317	T	C
24251514	T	A
24251561	GG	-
24252115	GAGA	-,GAGAGA
24252175	A	C
24252785	G	A,GTTTATTTATTTATTTA
24252789	A	G
24253167	T	A
24253322	A	G
24254019	T	C
24254064	-	A
24254145	T	C
24254390	-	T,TTT
24254391	T	G
24254548	T	A
24254606	AT	-
24254846	A	G
24255273	C	T
24255568	G	A
24255682	A	-
24255704	T	A

24255899	A	T
24256083	A	G
24257771	C	T
24257883	A	-
24258189	C	G
24258236	A	C
24258420	A	G
24258831	A	G
24258865	A	G
24258915	A	G
24258925	G	A
24259395	-	CCGATTT
24259397	-	T
24259398	G	C
24259442	G	C
24259509	T	C
24259561	G	A
24259568	GGGGT	-
24259616	T	A
24262729	C	T
24263824	G	A
24263919	C	T
24264059	C	T
24264581	G	A
24264635	-	GT
24265951	C	G
24266106	T	C
24266174	G	T
24266461	TTCT	-
24266619	T	C
24266650	-	G
24266732	-	TA
24266734	T	C
24266922	G	C
24266976	G	C
24266982	A	C
24267079	T	C
24267083	A	T
24267106	G	A
24267150	T	C
24267153	G	A
24267173	C	A
24267174	A	G
24267477	T	C
24267508	G	C

24268162	-	TTTT
24268166	G	T
24268383	C	G
24268666	G	A
24269504	-	GGCGGC
24269649	C	G
24270065	C	G
24270172	G	A
24271108	T	-
24271374	A	G
24271832	GAA	-
24271965	C	T
24272049	C	A
24272123	T	C
24272295	C	T
24272333	G	A
24272384	T	G
24272670	C	T
24272682	T	C
24272712	A	-
24272841	C	T
24273017	C	A
24273105	TG	-
24273110	G	C
24273121	ATAA	-
24273148	C	A
24273255	G	C
24273870	G	A
24273912	G	C
24274390	T	C
24274643	CTG	-
24275108	A	G
24275116	T	C
24275148	C	T
24275194	C	T
24275204	-	TTA
24275264	C	T
24275799	A	T
24275869	C	T
24276051	T	C
24276521	A	C
24276604	C	T
24276866	T	C
24276956	T	-
24277003	A	G

24277299	T	-
24277301	G	A
24277353	A	G
24277360	-	CCC
24277527	T	G
24277618	G	A
24277637	A	G
24277692	G	A
24277860	C	T
24277906	A	T
24277949	G	T
24278115	A	G
24278117	G	T
24278274	C	T
24278485	A	G
24278840	T	A
24278892	-	AGAG
24279324	G	T
24279373	A	T
24279403	AATC	-
24280924	A	-
24280926	G	T
24281210	A	-
24281444	-	TT
24281512	A	T
24282330	A	T
24282331	A	C
24282500	C	T
24282517	G	A
24282707	T	C
24282896	G	A
24283006	C	T
24283361	T	G
24283449	A	G
24283454	A	G
24283467	A	G
24284350	C	T
24284444	T	-
24284514	G	A
24284608	-	AA
24284618	T	C
24284723	A	T
24284753	A	G
24285046	T	C
24285073	G	A

24285148	A	G
24285313	A	G
24285317	A	G
24285358	T	C
24285375	C	G
24285422	A	C
24285434	T	C
24285502	A	T
24285537	T	A
24285544	A	G
24285648	-	CT
24285680	A	T
24285874	-	TCTT
24285885	AATA	-
24285912	A	T
24285956	T	C
24285957	G	A
24286156	AA	-,A
24286168	A	-
24286183	C	A
24286238	G	A
24286318	A	G
24286382	A	G
24286514	G	A
24286522	A	C
24286530	AGG	-
24286690	A	G
24286709	-	CA
24286721	G	A
24286826	A	C
24286885	A	C
24286939	G	C
24287122	T	C
24287138	T	-
24287141	A	-
24287156	T	C
24287666	G	C
24287870	T	G
24288034	G	T
24288077	A	T
24288154	A	G
24288369	G	A
24288532	G	-
24288537	G	C
24288614	A	G

24288814	T	C
24288919	A	G
24289007	A	G
24289119	G	T
24289158	-	AG
24289170	A	G
24289174	A	C
24289175	G	A
24289227	T	-
24289274	AGAG	-
24289351	A	C
24289354	-	A
24289371	A	G
24289415	A	G
24289428	C	A
24289582	C	T
24289998	G	C
24290082	T	C
24290123	G	A
24290174	T	C
24290386	G	A
24290573	G	T
24290859	G	A
24290975	A	G
24291664	G	A
24291973	A	C
24292019	G	A
24292304	G	T
24292521	A	G
24292900	A	C
24292922	TACT	-
24292994	AA	-
24293172	C	T
24293183	T	-
24293296	C	T
24293563	A	G
24293665	G	A
24293669	A	C
24294143	G	A
24294463	T	C
24294659	G	A
24295208	A	G
24296527	G	A
24296606	-	TTT
24296608	G	-

24296608	G	T
24296861	-	TGA
24297011	G	A
24297256	C	T
24297283	-	AAAAA
24297903	A	G
24297924	G	T
24298063	T	-
24298144	C	T
24298266	A	T
24298361	A	G
24298569	G	A
24298714	T	A
24298833	G	A
24298842	A	T
24298892	C	G
24299113	T	C
24299367	A	G
24299918	-	TTCG
24300642	G	A
24300952	TT	-
24301207	C	T
24301210	C	G
24301519	C	T
24301916	C	T
24303292	T	G
24303506	-	GA
24305189	A	G
24307735	-	G
24307818	C	-
24307819	C	A
24308399	A	G
24308440	G	A
24309310	C	A
24309750	A	G
24309755	G	T
24310208	T	A
24310385	T	C
24310785	G	T
24310799	T	C
24310802	G	T
24310808	G	T
24310850	T	C
24310854	G	A
24310886	G	A

24310899	T	C
24310943	C	A
24310956	C	A
24310966	C	A
24310970	C	A
24310978	T	A
24310982	G	T
24310988	G	T
24310990	G	T
24310992	G	T
24310994	G	T
24310996	G	T
24310998	G	T
24311649	T	C
24312333	-	TAA
24312489	A	T
24313115	C	T
24313120	-	TCCTT
24313132	A	C
24313136	C	T
24313138	C	T
24313141	C	T
24313147	C	T
24313619	C	G
24313679	G	T
24313682	T	-
24313997	T	C
24314127	C	-
24314375	A	G
24314437	-	C
24314554	T	A
24314557	CCTCC	-
24314564	T	A
24314566	-	AG
24314624	C	T
24314649	G	A
24314922	T	G
24315269	T	C
24315466	C	T
24315854	T	-
24315944	-	GTGCC
24315980	-	A
24316072	T	G
24316430	A	G
24317544	T	G

24317581	A	G
24317845	G	A
24318218	T	C
24318565	T	C
24318775	A	G
24319304	A	C
24319362	G	C
24319521	A	C
24319737	C	T
24319778	A	C
24319856	-	AA
24319988	C	T
24320105	A	G
24321971	G	A
24322108	-	TA
24322109	G	A
24322113	GG	-
24322125	-	GGAG
24322134	TT	-
24322139	G	C
24322141	G	-
24322143	T	-
24322189	-	TGTG
24322202	-	C
24322202	T	A
24322205	A	-
24322354	A	G
24322384	C	G
24322404	A	T
24322406	A	T
24322486	C	A
24322536	A	C
24322581	G	C
24322583	G	A
24322610	T	A
24322618	C	T
24322670	C	T
24322683	-	CT
24322719	G	A
24322807	A	C
24322855	A	G
24322864	C	A
24322974	A	G
24323002	T	G
24323039	T	C

24323076	C	T
24323314	-	TG
24323711	C	T
24323815	A	T
24324161	G	A
24324485	A	G
24324661	G	T
24325212	A	G
24325698	-	A
24325734	G	A
24325974	A	-
24326069	G	A
24326333	G	T
24326553	-	TGTG
24327618	AT	-
24327667	A	G
24328032	G	T
24328237	C	G
24328907	-	GTCCTGAGATTGA
24328953	C	-
24328987	C	T
24329000	A	-
24329197	-	A
24329286	G	A
24329617	A	-
24329720	G	A
24329767	T	C
24329890	C	T
24329965	G	A
24329972	-	T
24330235	G	A
24330700	G	A
24330994	-	CTCT
24330998	G	C
24331022	A	T
24331463	A	G
24331531	C	T
24331550	A	G
24331580	A	G
24332225	A	G
24332274	T	-
24332313	GAGAGAGAGA	-,GA,GAGAGAGA
24332349	G	C
24332406	C	A
24332723	G	A

24333312	G	A
24333380	C	T
24333467	T	C
24333582	-	TTTTTTT
24334189	C	T
24334389	-	TT
24334942	C	A
24334961	T	C
24335036	A	G
24335179	T	C
24335216	C	T
24335249	A	C
24335257	C	T
24335277	A	T
24335524	-	T
24335777	T	A
24335788	A	-
24335881	T	C
24335911	-	C
24335912	-	CG
24335935	G	A
24336014	C	T
24336074	T	C
24336316	T	C
24336543	T	C
24337030	G	A
24337058	A	G
24337520	C	T
24337579	ACACAC	-
24337628	G	A
24337765	C	A
24338339	C	A
24338433	T	C
24338532	-	ACAT
24339008	ATCTATCTATC	-
24339035	TCTATCATCTATCG	-
24339062	TATCTATT	-
24339069	TATC	-,TATCTATCTATC,TATCTATCTATCTATC
24339081	TATCTATCT	-
24339103	C	-
24339105	ATCATCATC	-
24339291	C	T
24339302	T	G
24339457	C	T
24339471	A	G

24339549	-	ATC
24340903	A	G
24340984	A	G
24341131	G	T
24342994	G	T
24342997	T	A
24343714	A	G
24344531	TC	-
24344683	-	AA,AAAA,AAAAA
24344770	C	T
24346389	A	G
24346727	-	TTTT
24347629	T	C
24347693	C	T
24347712	A	G
24347729	A	C
24347775	TCTCTCT	-
24347789	CTCTC	-
24347830	-	GTT
24347850	C	T
24348109	-	T
24348219	C	-
24348267	C	G
24348416	A	G
24348520	C	T
24348565	G	A
24348664	T	C
24348699	G	C
24348733	A	G
24349354	C	T
24349483	G	C
24349651	A	T
24349824	C	T
24349899	-	T
24350016	TCAG	-
24350308	C	G
24350421	C	T
24350442	T	C
24350485	T	C
24350777	AGAGAGAG	-,AG
24350844	C	T
24350886	C	T
24350907	A	G
24351222	C	-
24351276	A	C

24351366	G	T
24351639	A	T
24351643	TGATTATC	-
24351653	C	G
24351677	TTTTTT	-
24351723	T	-
24351730	A	-
24351738	T	G
24351755	A	T
24351878	G	T
24352137	T	A
24352388	G	C
24352546	T	C
24352760	-	A
24352846	T	C
24352863	T	C
24353115	A	G
24353165	C	A
24353170	C	T
24353181	C	G
24353396	G	A
24353657	A	C
24354018	G	A
24354103	C	T
24354207	G	A
24354446	A	G
24354604	A	-
24354829	C	T
24354951	A	G
24355094	C	T
24355541	G	A
24355695	A	G
24355990	C	T
24356197	C	T
24356221	C	T
24356617	C	T
24356911	A	T
24357076	-	A
24357508	G	A
24357879	C	T
24357896	T	C
24358040	A	G
24358401	T	G
24358554	C	T
24358621	A	T

24359117	C	T
24359156	G	C
24360075	A	G
24360172	C	T
24360497	-	G
24360898	A	G
24361479	GAGA	-
24361595	A	G
24361640	T	-
24361723	G	A
24361884	G	A
24361940	-	TT
24362417	-	T
24362627	A	C
24363161	A	G
24363186	-	A
24363212	G	A
24363320	TCTC	-,TCTCTCTCTCTC
24363514	T	-
24363655	TTT	-,T
24363826	G	T
24364008	A	G
24364019	-	A
24364035	ATGGAATA	-
24364049	T	C
24364052	T	C
24364053	A	G
24364204	T	C
24364379	A	G
24364419	A	G
24364543	G	C
24364684	A	G
24364812	C	T
24365024	T	A
24365062	T	A
24365107	TTG	-
24365181	C	T
24365602	-	AAAAAAAACAAAC
24365799	C	T
24365943	G	A
24366446	C	A
24366537	A	G
24366611	A	T
24366612	G	T
24366857	G	A

24366871	T	C
24366933	A	C
24367120	T	G
24367656	C	T
24367784	-	AAAA
24367793	C	A
24367937	T	-
24368258	A	G
24368393	A	-
24368503	A	G
24368509	G	T
24368664	AAATAAAT	-,AAATAAATAAAT
24368706	T	A
24368977	C	T
24368980	C	T
24369063	G	C
24369118	C	T
24369145	A	C
24369425	-	T
24369445	A	C
24369871	A	G
24370289	T	-
24370559	C	T
24371178	C	T
24371227	T	C
24371293	G	T
24371307	-	TCTCTTT
24371316	T	A
24372073	C	T
24372370	G	T
24372424	C	G
24372508	C	A
24372543	T	-
24372569	-	T
24372595	A	G
24372957	C	T
24372959	C	T
24373008	AGCCA	-
24373014	A	T
24373018	A	C
24373094	G	A
24373158	C	T
24373175	G	C
24373302	C	T
24373335	T	C

24373561	G	A
24373603	C	A
24373648	-	T
24373658	G	A
24373661	T	-
24373872	C	T
24373918	C	T
24374025	C	T
24374196	T	C
24374230	A	-
24374444	C	T
24374907	AACT	-
24375038	G	A
24375054	G	A
24375143	-	TTTC
24375200	-	TTCTAAGTTATTTA,TTCTTTCTTTCTTTC
24375203	A	C
24375236	AG	-
24375423	T	C
24375535	A	T
24375691	G	T
24375884	TGAGACAAGAAAA	-
24376511	-	A
24377023	TT	-
24377168	T	C
24377232	T	C
24377454	C	A
24377461	G	C
24377464	C	T
24377465	A	G
24377468	G	C
24377474	-	TTAAA
24377505	C	T
24377546	G	A
24377550	G	C
24377648	C	T
24377699	-	AA,A
24377708	-	A
24377722	-	G
24377800	G	C
24377952	T	C
24377965	G	A
24377969	T	G
24378051	C	T
24378103	T	C

24378172	A	G
24378173	G	T
24378202	-	AAAACTTCATTCA
24378942	-	TATCCCAGA
24379044	G	A
24379183	T	C
24379237	CAAA	-
24379242	AG	-
24379555	A	G
24379615	-	G
24379616	-	TAAAAAAA
24379617	C	A
24380160	G	T
24380228	C	T
24380394	C	G
24380448	T	A
24380507	G	A
24381044	T	C
24381069	T	C
24381126	C	T
24381237	C	A
24381255	C	T
24381266	C	G
24381526	G	C
24381721	TT	-
24381763	-	CGAGAG
24381764	C	CAGAGAG,G
24381781	G	A
24382025	-	T
24382063	CCA	-
24382069	C	-
24382072	C	A
24382074	GTG	-
24382080	AA	-
24382082	A	C
24382084	AAAA	-
24382090	A	C
24382165	-	T
24382281	A	C
24382309	T	C
24382453	-	TATATAC
24382458	T	A
24382461	T	A
24382494	AT	-
24382495	T	A

24382500	A	G
24382557	G	A
24382730	C	A
24382960	G	A
24383033	C	A
24383369	C	-
24383371	TGAATAAATAA	-
24383384	A	G
24383386	A	C
24383401	T	A
24383674	A	C
24383715	A	G
24384389	C	G
24384486	T	C
24384573	G	A
24384602	T	C
24385266	C	T
24385276	A	G
24385380	A	G
24385457	AAC	-
24385468	C	T
24385506	C	T
24385568	C	A
24385609	A	T
24385722	G	A
24385792	T	C
24385843	A	C
24386010	A	C
24386031	C	T
24386046	A	G
24386052	T	C
24386067	-	AGCCAA
24386110	C	T
24386115	G	T
24386170	A	T
24386289	G	A
24386360	T	A
24386784	G	A
24387041	G	A
24387106	G	A
24387134	A	T
24387175	A	G
24387307	T	A
24387518	A	G
24387529	A	G

24387578	A	C
24387620	CTAAAAGG	-
24387862	G	A
24388010	-	AA
24388254	-	TTG
24388255	-	GTT
24388375	TC	-
24388414	-	GCAA,GCAAGCAA
24388448	C	G
24388543	C	T
24388624	G	T
24388635	T	-
24388649	-	ATTT
24388687	G	T
24388694	-	AGAGAG
24388694	A	C
24388890	A	-
24389228	G	C
24389346	A	C
24389697	A	-
24389741	G	A
24389768	T	G
24390249	-	T
24390484	T	G
24390732	G	C
24390868	C	A
24390887	C	T
24391995	T	C
24392228	T	C
24392332	A	G
24392688	A	C
24392817	TATTAA	-
24393234	T	C
24393446	T	C
24393792	C	T
24393872	T	C
24394230	TGT	-
24394231	G	A
24394554	C	T
24397024	-	G
24397360	-	TATG
24397646	C	T
24397834	G	C
24397956	C	T
24397980	C	T

24398038	A	C
24398126	G	-
24398562	A	-
24399264	A	G
24399444	A	G
24399555	T	C
24399738	A	C
24399795	A	T
24400698	C	T
24400846	A	G
24401119	C	-
24401474	A	G
24401858	G	A
24402030	A	G
24402173	TTG	-
24402649	T	A
24402705	T	G
24402900	AT	-
24402933	G	A
24403153	A	G
24403261	TG	-
24403349	C	T
24403406	A	G
24403715	A	T
24404461	G	A
24404713	C	T
24405284	T	A
24405639	C	G
24405695	C	T
24405821	TCCA	-,TCCATCCA
24405939	T	C
24407109	C	A
24407207	A	T
24407208	A	C
24407230	T	-
24407334	A	G
24407516	C	T
24408087	-	T
24408162	C	T
24408265	T	C
24408270	C	T
24408479	AAGA	-
24408482	AA	-,A
24408982	A	G
24409280	A	C

24409365	T	C
24409434	C	T
24409454	C	T
24409480	-	TGAA
24409660	C	A
24410017	CTTT	-
24410079	G	A
24410120	G	A
24410173	A	G
24410255	A	G
24410384	C	T
24410431	C	T
24410453	C	T
24410553	-	G
24410614	A	G
24410762	T	A
24410971	G	A
24411225	T	A
24411553	T	C
24411586	A	T
24411682	TATGTATGCAAGTGTAGAG	-
24411737	G	A
24411900	A	G
24412011	C	T
24412040	G	C
24412431	T	C
24412705	-	TTT
24413042	T	C
24413329	TT	-,T
24413358	T	C
24413640	T	C
24414040	T	C
24414135	G	A
24414497	T	C
24414697	G	A
24414987	A	T
24414994	G	T
24415505	-	A
24415551	AGAG	-
24415556	G	C
24415643	C	T
24415663	C	T
24415854	C	G
24416021	T	C
24416030	C	T

24416231	TTTATTTA	-,TTTA
24416238	A	C
24416242	A	C
24416246	A	C
24416250	A	C
24416254	A	C
24416258	A	C
24416262	A	C
24416266	A	C
24416270	A	C
24416274	A	C
24416278	A	C
24417452	-	T
24417894	G	T
24418338	T	C
24418538	T	C
24418576	A	G
24418836	G	A
24418856	-	GGAGAGAGT
24419409	C	T
24419607	T	-
24419907	C	G
24419908	C	T
24419952	-	TGTCTC,TGTCTCTGTCTC,TGTTTCTGTCTCTGTCTCTG TCTC
24420079	G	A
24420110	A	T
24420230	-	AAGTCAGGAGCTGCCAA
24420603	T	C
24420808	A	T
24420830	C	T
24420859	G	A
24420864	A	-
24420923	C	T
24420999	C	T
24421106	G	A
24421283	T	C
24421357	T	C
24421452	ATGA	-
24421595	A	G
24421644	-	T
24421646	A	C
24421648	A	C
24421653	A	T
24421656	AGGA	-

24421682	G	A
24421734	G	T
24421840	AGAAAG	-
24421844	AGAG	-
24421956	T	C
24422086	T	C
24422316	C	T
24422366	T	C
24422394	T	G
24422431	G	A
24422553	A	C
24422571	C	G
24422958	-	AC
24422961	AG	-
24422961	A	C
24422962	G	C
24422965	A	G
24423006	T	C
24423104	G	A
24423234	T	G
24423375	G	A
24423919	AT	-
24423943	G	C
24424182	G	C
24424308	T	C
24424323	A	G
24424566	T	G
24424818	C	G
24424853	A	G
24424906	G	T
24425107	A	G
24425438	C	T
24425794	G	A
24425835	T	C
24425886	CTCTGTTTTGTATTTTTTTAATTTATTTTTTATTGGTGTT<br>CAATTTACTAACATACAGAATATCCCCCAGTGCCCGTC<br>ACCCATTCA	-
24426118	G	A
24426123	T	C
24426129	G	A
24426257	A	G
24426267	A	G
24426406	C	T
24426564	T	C
24427049	G	A

24427052	C	T
24427072	T	C
24427141	T	C
24427150	A	G
24427153	G	A
24427211	-	G
24427211	A	G
24427291	A	G
24427307	-	A
24427396	T	C
24427401	C	T
24427530	C	T
24427606	G	-
24427652	T	C
24427710	C	T
24427895	G	A
24428181	C	A
24428365	A	G
24428399	A	G
24428628	A	G
24428673	C	A
24428703	-	A
24428796	A	G
24429099	C	T
24429127	-	ATG
24429193	G	A
24429480	C	T
24429623	G	A
24429683	ATA	-
24429787	C	T
24430035	C	T
24430093	A	G
24430309	C	T
24430649	C	T
24430933	A	G
24430970	A	G
24431641	T	A
24431859	C	T
24432113	-	AATA
24432330	C	T
24434006	C	T
24434011	T	C
24434296	C	T
24434419	T	C
24434421	A	G

24434442	CAGA	-
24435009	G	T
24435184	A	G
24435493	C	T
24435840	C	T
24436038	T	C
24436065	G	T
24436088	T	C
24436091	G	C
24436133	A	G
24436393	C	G
24436548	G	A
24436575	T	C
24436732	A	-
24436746	A	C
24437043	T	C
24437071	G	A
24437142	-	CATAC
24437277	G	T
24437295	T	C
24437306	T	C
24437368	T	G
24437427	T	G
24437490	C	A
24437664	G	A
24437666	C	T
24437804	C	-
24437826	T	C
24437831	G	A
24437983	C	T
24439204	TCCATCCA	-
24440084	A	G
24440355	G	T
24440366	GTGT	-
24440408	G	T
24440761	T	C
24440762	G	A
24440796	G	T
24440833	C	T
24440863	T	C
24441143	C	T
24441273	A	G
24441309	A	G
24441336	C	-
24441342	G	A

24441688	G	A
24441941	G	A
24442004	G	C
24442093	G	T
24442152	A	G
24442225	G	A
24442332	C	G
24442480	G	T
24442486	C	A
24442501	C	A
24442506	C	A
24442511	C	A
24442516	C	A
24442521	C	A
24442526	C	A
24442746	T	C
24442846	G	A
24442931	T	C
24442938	G	A
24443171	G	A
24443242	T	G
24443338	-	C
24443520	C	T
24443598	T	C
24443643	G	A
24443716	T	C
24443864	C	G
24443978	AA	-
24444354	A	G
24444360	-	T
24444419	G	A
24444421	-	AAAAACA,A
24444425	CT	AT,C
24444426	T	C,A
24444428	-	AAAC
24444434	C	-
24444438	C	A
24444508	T	C
24444551	G	A
24444587	-	CACT
24445606	-	ATG
24445676	A	G
24445877	A	C
24446944	G	A
24446964	G	A

24447016	C	T
24447139	A	T
24447309	A	G
24447350	G	A
24447363	G	A
24447425	A	T
24447500	G	A
24447503	C	G,T
24447604	G	T
24447650	C	T
24447739	AATTTAAATAAATTAATTTAATAAATTAATAATTTATTAAATA	-
24447834	A	T
24447837	-	A
24447859	A	T
24447879	A	C
24447922	A	G
24448004	TGAGCTACCCCAGTTAG	-
24448022	T	G
24448106	T	A
24448657	G	A
24448746	A	C
24448845	G	T
24448890	A	G
24448935	A	T
24449191	CACA	-
24449195	-	TGTG
24449332	A	T
24449454	T	C
24449455	T	C
24449504	C	A
24449536	C	T
24449551	T	C
24449553	C	A
24449595	G	A
24449682	-	TA
24449752	-	C
24449887	C	G
24450067	C	T
24450242	G	A
24451061	G	T
24452102	-	CCATTTGACTC
24452594	C	T
24452637	C	T
24452727	T	A

24452769	TAAG	-
24452773	TAAA	-
24452798	AAATAAATAAATAAATAAA	-
24452802	AAATAAATAAATAAA	-
24453058	A	C
24453153	-	A
24453207	T	C
24453272	-	GCTCCACCTCCG
24453308	T	C
24453336	-	TT
24453344	C	G
24453366	C	A
24453368	A	G
24453432	G	-
24453434	GAGAGAGAGAG	-
24453467	G	C
24453554	C	T
24453742	T	G
24453791	TG	-
24453793	-	AAA
24453942	C	T
24453946	A	G
24454206	C	T
24454860	G	A
24454994	A	G
24455113	C	A
24455573	C	A
24455577	C	T
24455652	-	AAATAAATAAAT
24455733	GG	-
24455836	A	G
24456060	A	-
24456171	G	C
24456250	T	C
24456271	G	C
24456482	G	A
24456586	G	C
24456723	AT	-
24456724	T	-
24456826	T	-
24457079	C	T
24457268	A	G
24457304	A	G
24457381	G	A
24457446	G	A

24458008	T	A
24458062	T	C
24458106	C	T
24458481	T	-
24458622	C	T
24458700	T	G
24458701	A	C
24459471	C	A
24459645	C	T
24459827	C	T
24459929	C	T
24459941	A	G
24459993	A	G
24460141	-	TCAT
24460411	T	G
24460895	TA	-
24460902	G	A
24461105	G	A
24461121	A	G
24461692	G	A
24461722	T	C
24461822	T	-
24461824	TA	-
24461908	T	C
24461935	G	A
24461960	C	T
24461985	A	T
24462679	GC	-
24462803	C	T
24462850	A	G
24462851	G	A
24462942	A	G
24463122	-	T
24463157	-	GAGA
24463245	C	T
24463247	C	T
24463281	T	C
24463505	T	G
24463527	A	G
24463581	C	T
24463601	G	A
24463623	T	C
24463646	T	C
24463654	T	-
24463722	T	C

24463725	C	T
24463752	C	T
24463869	C	A
24463907	G	A
24463944	T	C
24464030	T	A
24464040	T	-
24464286	C	A
24464287	T	G
24464290	C	T
24464305	A	T
24464338	C	T
24464424	T	C
24464428	G	T
24464473	T	G
24464499	G	A
24464523	C	T
24464539	A	G
24464563	C	T
24464573	C	T
24464587	A	G
24464588	C	T
24464606	C	T
24464610	T	C
24464667	C	T
24464712	C	A
24464717	-	A
24464765	T	C
24464843	T	C
24464863	G	T
24464868	G	T
24464931	-	A
24465170	T	C
24465171	G	A
24465183	-	TCTTTTTTTTT
24465224	T	C
24465242	-	TATTTATT,TATTTATTTATT
24465281	-	T
24465317	GAGAGACACA	-
24465390	G	A
24465401	C	T
24465434	A	G
24465451	T	C
24465452	G	C
24465466	C	G

24465488	A	C
24465585	C	T
24465647	C	A
24465713	A	T
24465760	C	T
24465833	T	C
24466008	C	T
24466033	-	TTC
24466037	G	T
24466040	G	T
24466047	T	C
24466128	A	C
24466136	A	G
24466389	-	C
24466393	G	A
24466558	-	T
24466776	T	A
24467056	A	G
24467243	A	G
24467686	T	A
24467695	-	TA
24467845	T	C
24468121	C	T
24468505	C	T
24468567	C	T
24469637	T	G
24469685	C	T
24469760	C	T
24470005	A	G
24470666	G	A
24471171	G	C
24471231	A	T
24471251	C	A
24471463	CCTTCTTT	-
24471464	C	T
24471802	A	G
24472104	T	A
24472216	G	A
24472562	G	T
24472930	T	A
24473073	A	T
24473265	G	A
24473537	T	C
24473644	T	A
24473780	C	G

24473883	T	C
24473947	A	C
24474030	TAATAATAA	-,TAATAA
24474225	T	G
24474391	C	T
24474932	T	C
24475031	T	C
24475135	G	A
24475313	TTTTTT	-
24475453	A	C
24475591	A	G
24475782	G	A
24476062	G	C
24476226	G	A
24476656	T	C
24476716	G	A
24476767	C	T
24476863	G	A
24476870	T	C
24476991	A	G
24477156	C	T
24477192	C	T
24477536	-	C
24478134	C	T
24478191	T	C
24478356	T	A
24478515	T	-
24478517	G	A
24478519	T	C
24478521	G	T
24479293	C	T
24479706	-	A
24479775	G	A
24479789	G	A
24479967	G	A
24480116	C	T
24480124	T	C
24480328	G	A
24480341	AG	-
24480345	A	T
24480368	C	G
24480369	A	G
24480390	G	-
24480427	A	T
24480459	G	A

24480475	G	T
24480602	G	A
24480837	A	G
24480903	T	-
24480997	G	A
24481021	G	A
24481044	-	TT
24481044	C	T
24481046	C	T,CTTT
24481120	G	A
24481157	C	G
24481369	T	C
24481424	C	T
24481590	A	G
24481772	T	-
24481931	C	T
24481976	-	G
24482006	T	C
24482048	C	-
24482066	T	A
24482068	G	A
24482100	T	A
24482146	A	G
24482242	A	T
24482291	A	T
24482371	C	T
24482421	G	A
24482451	T	C
24482470	A	G
24482483	C	G
24482493	T	C
24482535	T	A
24482675	T	G
24482690	G	T
24482791	A	G
24482822	A	C
24483006	AAAAATATATATATATATATATATATATATAT	-
24483098	T	C
24483114	T	A
24483209	C	T
24483214	C	T
24483246	T	C
24483265	-	TG
24483296	A	-
24483425	C	A

24483453	G	C
24483639	G	C
24483720	TTTTTT	-,TTTTT
24484353	G	T
24484591	C	G
24484592	C	A
24484605	C	T
24485155	A	C
24485183	G	C
24485206	A	G
24485241	C	A
24485249	A	G
24485342	-	TTTATTT
24485348	-	TTATTTTTAAGAATTCTTTTTTTATTTTTTTTTTTATTTT<br>TTATTTTTTTT
24485477	G	T
24485519	C	T
24485556	T	C
24485690	C	G
24485784	G	A
24486131	G	T
24486190	G	C
24486229	C	T
24486257	G	A
24486294	G	A
24486366	G	A
24486868	A	G
24486923	A	T
24488029	A	G
24488520	A	C
24488615	T	A
24488659	T	C
24489222	G	T
24489258	-	A
24489358	T	A
24489517	G	A
24489928	A	G
24490286	T	C
24490660	A	-
24490720	C	T
24490764	T	C
24491208	A	T
24491395	T	G
24491489	T	A
24491661	T	A

24491766	A	G
24491989	C	T
24492160	T	C
24492200	A	G
24492383	G	A
24492617	A	G
24492741	G	A
24493784	C	G
24494087	C	A
24494177	A	C
24494225	T	C
24494256	A	T
24494747	C	T
24494978	T	C
24495061	A	G
24495402	C	T
24496144	T	C
24496188	A	C
24496338	A	T
24496393	T	C
24496543	C	T
24496604	C	T
24496875	A	G
24496908	-	TG
24496945	-	A
24497011	A	G
24497299	A	G
24497818	G	A
24498233	C	T
24498861	A	G
24499336	-	A
24499561	G	A
24499726	C	A
24499765	T	C
24500127	G	A
24500481	G	A
24500606	T	-
24501055	C	T
24501177	G	A
24501411	A	T
24501964	-	T
24502719	T	-
24502991	T	A
24503817	T	C
24504015	T	C

24504099	T	-
24504867	GTTT	-
24504886	C	T
24505071	T	C
24505075	T	C
24505526	C	A
24505554	G	C
24505838	T	C
24506153	C	A
24506177	G	C
24506442	T	C
24506708	A	C
24507272	C	T
24507311	A	T
24507339	C	T
24507492	A	G
24507536	T	C
24507547	A	G
24507655	-	A
24507872	G	A
24507966	T	A
24508188	-	A
24508475	T	C
24508583	G	A
24508585	T	C
24508783	C	T
24509004	C	G
24509034	-	TT,TTTT
24509119	T	C
24509305	T	-
24509674	A	T
24509676	A	T,ATT
24510127	C	T
24511486	C	T
24511680	A	G
24511986	T	A
24512068	T	-
24512278	C	T
24512286	C	T
24512378	C	T
24512420	C	T
24513080	C	A
24513777	G	-
24516271	T	C
24516314	T	C

24516551	A	G
24517411	G	A
24517413	A	G
24517934	-	TTTTTTATTCATT
24517935	T	C
24519127	T	A
24519186	C	T
24519228	G	A
24519237	C	T
24519400	T	C
24519422	A	G
24519511	A	G
24519526	T	C
24519566	A	G
24520134	T	-
24520379	T	-
24520437	T	C
24520948	GG	-
24520981	G	A
24521142	A	G
24523279	T	A
24523350	T	A
24523535	-	TACACACACAC,TACACACACACAC
24523550	G	C
24523574	C	-
24523619	T	C
24523977	A	G
24524492	G	A
24525948	G	T
24526212	A	C
24526659	A	G
24526717	C	T
24527036	A	G
24527286	A	T
24527287	C	T
24527346	-	TTAT
24528014	T	G
24528465	A	T
24528915	-	GTGTGT,GTGTGTGT
24529036	G	A
24529345	T	-
24529350	T	A
24529351	T	A
24530020	C	T
24530207	A	G

24530359	T	C
24530943	A	G
24531392	A	-
24531533	T	C
24531559	C	T
24531915	T	C
24532057	-	T
24532203	A	G
24532233	A	G
24532240	-	AG
24532311	T	G
24532322	-	GG
24532401	AGAGAGAGAAATAC	-
24532468	G	C
24532716	A	-
24533017	A	G
24533181	T	C
24533203	A	T
24533204	T	C
24533308	A	G
24533349	T	C
24533510	T	C
24533564	A	C
24533713	A	G
24534126	C	T
24534150	C	T
24534153	G	C
24534162	-	T
24534222	C	T
24534278	G	A
24534395	-	CT
24534592	G	A
24534606	-	GTTTTTGTTTTT
24534679	G	A
24534916	TGT	-
24534918	-	TT
24534920	T	A
24535359	A	G
24535431	T	C
24535521	G	T
24535917	C	T
24535953	C	T
24536116	A	G
24536126	-	CTT
24536160	TTCTTCTTCTTCTTCTTCTTT	-

24536221	-	GG
24536223	G	A
24536249	AGA	-
24536402	C	T
24536432	C	T
24536530	T	C
24536630	A	T
24536787	C	T
24536971	A	G
24537023	C	A
24537313	A	G
24537738	T	A
24538373	T	C
24538670	A	G
24538769	T	A
24539281	T	C
24539282	C	G
24539410	C	T
24539527	G	A
24539589	A	G
24539664	A	T
24540024	-	AC
24540255	A	G
24540433	C	T
24540452	-	TT
24540647	T	-
24540684	G	T
24541070	T	-
24541461	G	A
24541491	G	A
24541638	A	T
24542065	T	C
24542134	T	G
24542439	-	C
24542498	-	A
24542519	G	T
24542521	-	AG
24542562	-	A
24542697	T	C
24542822	T	G
24542826	G	A
24543068	C	T
24543197	G	A
24543237	C	T
24543276	C	T

24543404	C	A
24543412	C	A
24543485	T	A
24543622	C	T
24543699	A	G
24543981	-	AAAT
24543995	A	-
24544036	A	G
24544169	G	A
24544335	AG	-
24544546	A	G
24545099	G	A
24545113	T	G
24545122	C	G
24545141	C	A
24545252	C	T
24545371	A	G
24545412	G	T
24546205	C	A
24546254	T	C
24546418	T	-
24546489	T	C
24546662	A	C
24546746	T	C
24546763	-	TTG
24546835	CCC	-,CC,CCCC
24546911	A	G
24547342	G	A
24547343	T	C
24547776	-	T,TT
24547986	G	A
24548097	C	G
24548256	T	C
24548560	-	AAA
24548617	C	T
24548716	A	C
24548720	T	C
24548750	C	T
24549051	C	G
24549136	A	G
24549626	C	T
24549873	C	A
24550107	T	A
24550130	G	A
24550189	T	C

24550190	G	A
24550235	C	T
24551375	-	GCAAG
24551832	A	G
24552211	C	G
24552213	G	C
24552410	A	G
24552813	T	C
24553240	-	AAATGGGA
24553241	T	G
24553437	ATG	-
24553525	-	AT
24554016	C	T
24554056	A	T
24554082	G	T
24554089	A	T
24554201	A	T
24554252	A	G
24554288	T	C
24554350	-	A
24554696	C	T
24554814	C	T
24554959	A	G
24555465	T	A
24555489	A	G
24555920	C	T
24556332	A	G
24556545	T	C
24556549	C	T
24556757	G	T
24556811	A	G
24556969	C	G
24556978	G	A
24557143	A	T
24557267	A	G
24557268	T	C
24557274	G	A
24557338	C	T
24557771	T	C
24558013	T	G
24558193	A	G
24558595	G	A
24558867	A	G
24559094	T	C
24559707	G	A

24559862	C	T
24559881	T	C
24559961	G	T
24560039	G	A
24560198	T	G
24560321	C	T
24561344	AT	-
24561389	C	T
24561441	ATATATAC	-
24561448	CG	-
24561448	C	T
24561452	-	TA
24561453	A	G
24561472	TA	-
24561479	-	ATATATATA
24561698	A	T
24562339	A	C
24562519	T	A
24562521	T	C
24563275	A	G
24563511	TGGATCCATG	-
24563522	C	A
24563523	C	A
24563525	T	G
24563532	T	C
24563775	AAGAGGACTATAC	-
24563903	T	C
24564013	C	G
24564059	A	G
24564079	A	G
24564155	CTATTTT	-
24564362	C	T
24564487	A	G
24564560	T	C
24564586	G	T
24564614	T	C
24564810	A	C
24565161	C	A
24565206	A	T
24565217	T	C
24565230	A	G
24565367	T	C
24565435	G	A
24565605	G	A
24565618	G	A

24565931	C	T
24566091	C	T
24566142	C	T
24566165	C	T
24566166	A	G
24566190	G	A
24566197	A	T
24566259	A	G
24566262	A	T
24566382	G	C
24566386	G	A
24566436	C	T
24566468	C	G
24566759	A	T
24566841	A	G
24567224	T	C
24567326	T	G
24567863	C	T
24568071	A	C
24568254	A	G
24568517	G	C
24568527	G	A
24568530	C	T
24568553	G	T
24568644	G	A
24568899	A	G
24568910	G	A
24568981	C	T
24568989	C	T
24569015	C	T
24569029	C	T
24569092	G	C
24569216	CCCAGAGAT	-
24569232	G	A
24569249	T	C
24569309	G	A
24569517	A	G
24569558	G	A
24569620	T	C
24569753	C	T
24569831	G	A
24569915	-	T
24569941	G	A
24569959	-	TTTA
24569978	-	CA

24569997	-	AGAG,ATAGAGAG
24570066	G	A
24570079	T	C
24570080	C	T
24570107	G	A
24570120	G	A
24570134	G	C
24570164	AA	-
24570193	T	C
24570313	C	-
24570319	A	C
24570954	TGT	-,T
24570956	-	TTTT
24570969	C	T
24571164	T	A
24571338	A	-
24571345	T	C
24571679	C	A
24571680	T	C
24571847	C	T
24571910	T	C
24572053	A	G
24572237	A	T
24572269	A	G
24572404	A	T
24572501	C	T
24572976	G	C
24573074	C	T
24573125	T	C
24573850	G	A
24573892	G	A
24574626	T	C
24574642	T	A
24574667	G	A
24574721	G	A
24575111	T	G
24575441	T	C
24575906	A	G
24576303	-	T
24576634	-	T
24576946	-	T
24577022	A	G
24577038	G	A
24577081	G	T
24577127	C	T

24577129	A	T
24577180	G	A
24577378	G	C
24577502	-	G
24577824	G	A
24578317	-	T
24578399	T	C
24578491	C	A
24578593	C	G
24579203	A	G
24579240	C	-
24579459	G	A
24579532	-	TATTTATTTATTTATC
24579666	C	A
24579680	G	A
24579816	A	C
24580051	T	C
24580052	G	A
24580068	G	A
24580072	T	A
24580073	T	A
24580077	T	C
24580108	T	C
24580110	C	G
24580127	-	T
24580194	G	A
24580274	T	C
24580320	A	G
24580398	T	C
24580443	A	G
24580482	-	CC
24581254	C	T
24581449	T	C
24581581	C	T
24581611	G	A
24581852	A	T
24582405	G	A
24582792	G	A
24583198	G	A
24583236	C	T
24583264	GTATT	-
24583397	G	A
24583471	T	C
24583560	A	G
24583780	G	A

24583882	G	A
24583963	GGTCACCCTCCAGA	-
24584032	T	A
24584079	A	G
24584100	TA	-
24584232	C	T
24584355	C	T
24584367	-	T
24584431	-	A
24584486	C	T
24584580	G	A
24584719	G	A
24584783	C	T
24584795	A	C
24585010	A	-
24585152	T	A
24585247	G	A
24585263	A	G
24585267	C	T
24585297	C	T
24585378	-	CTCTCT
24585419	-	A
24585483	C	T
24585559	T	A
24586283	A	G
24586285	C	A
24586475	C	T
24586501	T	-
24586825	T	A
24586879	A	G
24587023	G	A
24587055	C	T
24587147	T	C
24587220	T	C
24587265	T	C
24587299	T	-
24587391	G	A
24588199	A	G
24588294	C	T
24588432	C	G
24588460	A	G
24588499	T	A
24588500	G	A
24588705	T	G
24588971	G	C

24588980	-	A
24589072	A	G
24589131	A	T
24589184	A	C
24589404	G	T
24589846	C	T
24589913	A	G
24590079	T	C
24590188	G	A
24590208	T	C
24591025	C	T
24592509	A	G
24592783	G	A
24593085	G	A
24593220	-	GGAATGTAAAGATAATTT
24593793	T	-
24593837	-	AC
24593842	G	C
24594752	T	C
24594763	T	C
24594813	G	A
24594990	A	G
24595027	C	G
24595220	C	-
24595410	G	A
24595726	-	CCAAA
24595728	T	A
24595730	G	A
24596495	G	A
24599548	T	-
24600487	G	A
24600602	-	AGCTTCATTAATTTTATTG
24601015	A	G
24601423	-	A
24601502	A	-
24601778	GG	-
24601905	T	C
24602368	T	A
24603675	C	T
24603868	G	A
24603904	A	-
24604233	-	AGAG
24604558	G	A
24605055	-	TTTA
24605471	A	G

24605559	C	A
24605860	G	A
24606252	A	G
24606543	T	C
24607423	C	T
24607426	-	GAAG
24607729	G	A
24608485	G	T
24609079	-	TGTGTG
24609191	T	C
24609999	TTC	-
24610186	A	G
24610718	G	A
24612572	C	T
24612698	T	C
24612770	-	AG
24612883	A	G
24612966	C	A
24613160	G	A
24613256	A	C
24613420	C	T
24613592	AAAAA	-,A
24614201	A	T
24614500	T	C
24614720	T	C
24614776	C	T
24614829	T	C
24614955	G	T
24614969	A	T
24615493	T	A
24615497	A	T
24615499	A	T
24615663	T	A
24615718	T	A
24615776	C	A
24616114	T	A
24616124	A	G
24616212	G	A
24616234	G	C
24616440	C	T
24616622	C	T
24616971	C	T
24617344	A	-
24617411	T	C
24617530	-	T

24617777	C	T
24617809	G	T
24618583	T	C
24618827	-	T
24618947	G	A
24619124	T	C
24619169	A	C
24619373	A	G
24619504	A	G
24619659	G	A
24619735	C	T
24619795	G	A
24619806	C	G
24619856	TGGGGTAGT	-
24619864	-	AAC
24619865	G	C
24619868	GGGGGTGGG	-
24620077	T	C
24620131	G	A
24620146	C	T
24620542	A	T
24620827	C	A
24621035	TTTCTT	-
24621195	T	C
24621197	-	T
24621409	A	T
24621916	G	A
24622269	A	G
24622436	CAG	-
24622564	G	A
24622732	A	G
24622874	T	G
24623218	C	T
24623489	C	T
24623650	A	G
24623674	C	T
24623994	G	-
24623995	A	T
24624277	A	C
24624328	AGAGAG	-
24624608	C	T
24624757	T	C
24625045	A	G
24625282	C	A
24625880	A	T

24626774	A	T
24626788	C	T
24626789	G	T
24627034	T	C
24627090	C	A
24628212	A	G
24628230	C	G
24628430	-	G
24628719	C	T
24632148	A	G
24632536	T	-,TT
24632778	A	G
24633481	C	T
24634034	-	TTT
24634047	C	T
24634843	C	T
24635150	C	G
24635366	G	C
24635633	G	A
24635961	AAC	-
24635989	-	ACG
24635994	A	G
24636317	C	T
24636414	G	A
24636416	A	G
24636646	C	T
24636678	C	G
24637117	-	TT
24637176	AAC	-
24637217	T	C
24637281	A	G
24637338	T	A
24637415	C	T
24637479	C	A
24637492	G	A
24637494	A	G
24637542	C	T
24637547	C	T
24637818	C	G
24637861	T	A
24637865	A	G
24637876	G	-
24638096	T	C
24638340	TTGTAAAATATAT	-
24638347	-	ATATAT

24638357	-	AC
24638416	A	C
24638577	TACT	-
24638707	T	C
24638720	G	A
24639354	T	A
24639356	T	G
24639385	G	A
24639431	G	A
24639489	-	G
24639836	C	T
24639994	G	A
24640056	-	T
24640078	TTC	-
24640242	T	C
24640305	G	A
24640360	T	C
24640372	G	A
24640419	ATATAT	-,AT
24640519	A	G
24640599	C	T
24640635	A	-
24640702	G	A
24640785	T	C
24640888	A	G
24640939	T	C
24641346	A	T
24641451	A	G
24641454	C	T
24641485	T	C
24641489	G	A
24641556	TT	-
24641575	C	T
24641660	AC	-
24641667	C	A
24641767	-	T
24641870	A	G
24641887	A	G
24641918	G	C
24641949	C	G
24642278	C	T
24642355	T	C
24642591	A	G
24642699	C	T
24642727	T	C

24642773	T	C
24642839	A	G
24642977	G	A
24643000	A	G
24643042	T	A
24643100	A	G
24643373	A	C
24643441	A	T
24643547	T	C
24643621	G	A
24643646	-	G
24643650	-	G
24643653	-	TG
24643902	C	T
24644102	-	TGAA
24644174	AAAAA	-
24644196	T	A
24644263	T	C
24644717	G	A
24645060	T	C
24647086	CTATC	-
24647100	A	T
24647101	C	A
24647105	A	T
24647107	A	T
24647108	A	T
24647860	-	TCAAGCA
24647862	-	CAA
24648123	-	A
24653982	C	T
24654646	G	T
24654673	T	C
24654845	A	T
24654869	C	A
24654899	A	G
24655280	T	C
24656073	T	C
24656114	G	A
24656611	A	G
24656662	C	T
24656665	C	G
24657005	G	A
24657117	-	T
24657149	T	A
24657162	G	T

24657170	T	C
24657172	G	A
24657229	G	A
24657236	T	C
24657247	G	C
24657265	CTCTCTCT	-,CT,CTCTCTCTCTCT
24657299	T	C
24657316	A	T
24657323	A	T
24657392	G	A
24657497	T	A
24657629	A	-
24657691	T	C
24659070	C	T
24659895	AC	-
24660023	T	C
24660476	-	AG
24660582	G	A
24660686	A	T
24660714	G	C
24660727	C	T
24660929	C	T
24661031	T	C
24661218	A	T
24661939	T	G
24662080	C	T
24662115	A	G
24662347	C	A
24662518	A	G
24662580	G	A
24663057	C	G
24663084	CCTTCCTTCCTTCCTTCCTTCCTTCCTTCCTTCCTTCCTCCCTCCCTCCCTCCCTCCCTCCCTCCCTCCCTCCCTCCCTC	-
24663446	C	T
24663607	G	A
24663698	C	T
24664444	T	G
24664684	G	T
24665238	T	C
24665386	G	A
24665557	-	TAGAAACTGAACG
24665828	C	T
24665829	G	T
24665908	T	-

24666134	C	T
24666247	-	AA
24666523	C	A
24666888	G	A
24666990	G	A
24667016	TT	-
24667046	AAAAGTAATTCTTTTAAAAAAAAAAAAAAAAAAA	-
24667124	T	A
24667137	A	G
24667160	G	A
24667165	A	G
24667191	-	CT
24667739	-	C
24667740	T	A
24667843	A	T
24667986	A	T
24668284	T	C
24668321	G	A
24668430	T	C
24668444	A	G
24668607	A	G
24668612	G	T
24668628	A	T
24668639	A	G
24668665	AAATAAAT	-,AAAT
24668706	C	T
24668707	A	G
24668716	A	T
24668811	T	C
24668833	A	G
24668999	T	C
24669042	C	T
24669054	T	C
24669375	-	T
24669464	TCTT	-
24669584	G	C
24669746	-	A
24669994	T	C
24670052	C	A
24670104	A	G
24670388	A	C
24670715	TTT	-,T
24670719	T	C
24670721	T	C
24671017	G	T

24672567	C	T
24672694	-	A
24673174	-	T
24673551	-	TGTAGACTG
24673584	C	T
24673810	A	T
24673860	G	T
24673893	T	G
24674083	A	G
24674614	G	A
24674821	G	T
24674990	C	G
24675165	G	C
24675412	C	G
24675621	A	G
24676040	G	A
24676182	A	C
24676361	A	G
24676436	C	T
24676538	G	A
24676713	C	T
24676819	C	G
24676973	G	T
24677018	-	T
24677046	G	C
24677152	C	A
24677179	G	A
24677351	T	C
24677426	G	T
24678687	A	G
24679721	T	C
24679738	A	G
24679772	T	C
24679815	T	C
24679862	G	C
24679938	T	A
24680228	C	A
24680338	G	A
24680389	A	C
24680830	A	G
24680897	T	A
24680918	C	T
24681087	T	C
24681468	G	A
24682089	T	G

24682165	G	A
24682902	CT	-
24683794	TATATTAA	-
24683860	T	G
24683880	G	A
24683990	C	T
24684189	A	G
24684293	C	T
24685240	G	A
24685577	G	C
24685795	A	G
24685888	T	G
24685931	ATGAGT	-
24686184	A	G
24686370	C	A
24686397	T	C
24686423	ACTG	-
24686679	-	A
24686820	A	G
24686929	T	A
24687034	ACT	-
24687170	T	G
24687175	G	A
24687892	G	A
24688065	C	T
24688143	A	G
24688939	C	T
24689095	A	T
24689096	T	C
24689455	T	A
24689681	G	A
24689741	G	T
24689764	T	C
24689774	A	G
24690004	T	C
24690339	C	T
24690343	A	T
24690374	-	AA,AAA
24690605	-	TCTT,TCTTTCTT
24690650	-	TTC,CTTTTTC
24690945	T	-
24690961	C	T
24691145	C	T
24691324	T	C
24691370	C	T

24691481	G	T
24691494	A	-
24691501	ATTTTTTTT	-
24691502	T	-
24691539	A	-
24691543	C	-
24691550	-	T
24691557	-	C
24691561	-	C
24691565	-	C
24691569	-	C
24691572	T	A
24691597	A	-
24691598	AT	-
24692097	A	G
24692429	C	T
24692431	G	A
24692614	A	G
24692657	T	C
24692711	-	T
24692870	T	A
24692873	T	C
24692874	G	A
24693108	C	T
24693170	C	T
24693171	A	G
24693323	G	A
24693548	C	T
24693634	C	T
24693671	A	G
24693819	-	TGTG
24693880	A	C
24693917	A	G
24694181	C	T
24694186	T	C
24694516	T	G
24694620	A	T
24694626	-	T
24694645	-	T
24694726	A	-
24694764	T	C
24694765	C	T
24694806	G	A
24694886	T	-
24694982	A	G

24695047	A	G
24695407	C	T
24695436	G	T
24695572	ACAC	-
24695828	ATTC	-
24695831	C	-
24695833	TTCATTCATTC	-
24695847	C	A
24695851	C	A
24695855	C	A
24695859	C	A
24696704	G	A
24696770	C	T
24697228	T	C
24697342	-	T
24697364	T	G
24697502	G	A
24697539	G	A
24697676	G	T
24697827	C	T
24698146	A	G
24698810	G	A
24698997	C	T
24699041	C	A
24699082	G	C
24699189	C	T
24699557	C	T
24699569	G	A
24699577	T	A
24699772	AT	-
24699788	G	A
24699930	C	T
24699961	C	T
24700078	A	G
24700203	G	C
24700220	G	-
24700243	G	A
24700317	C	T
24700403	A	G
24700462	-	T
24700536	C	T
24700711	T	A
24700819	T	C
24701081	C	A
24701471	C	T

24701570	G	T
24702274	C	T
24702723	C	G
24702837	T	C
24702894	A	G
24703362	-	T
24703441	-	TC
24703552	T	C
24703565	C	T
24703884	C	-
24703885	C	T
24703915	G	C
24703941	A	G
24703947	G	A
24704202	G	A
24704234	A	T
24704335	-	T
24704369	C	A
24704386	T	C
24704470	T	C
24704510	G	A
24704538	T	C
24704594	C	A
24704616	T	C
24704642	G	A
24704790	G	A
24705317	T	C
24705324	G	A
24705492	C	T
24705493	C	G
24705510	GAGAGAGAGAGA	-,GAGAGAGA
24705529	AGAGAGAGAC	-
24705554	G	A
24705577	G	-
24705599	C	T
24705620	A	G
24705754	G	A
24705836	C	T
24706137	A	T
24706446	T	A
24706600	A	G
24706657	C	G
24707185	G	T
24707686	C	T
24707690	A	G

24708181	G	A
24708410	G	A
24708519	C	T
24709942	G	T
24710243	A	G
24710293	-	AA
24710559	-	A
24710616	-	CTACT
24710788	C	T
24710885	C	T
24711604	C	A
24711846	G	A
24712680	G	A
24712727	T	A
24713452	G	A
24713508	C	T
24713617	T	C
24713667	T	C
24713710	G	A
24713780	T	C
24713833	C	T
24714933	G	A
24715050	G	A
24715051	C	A
24715517	A	G
24715578	A	G
24715892	C	G
24715896	T	C
24716009	T	C
24716056	T	C
24716339	A	G
24716456	T	C
24716697	G	-
24716749	C	T
24716839	A	T
24716855	G	C
24716857	C	A
24716884	A	C
24716888	G	A
24716958	A	C
24716965	-	A
24716969	G	T
24716974	G	A
24716985	-	T
24716989	G	A

24717004	-	T
24717007	-	T
24717012	AT	-
24717455	-	CTTTTTTTT
24717479	C	T
24717629	G	T
24717677	A	T
24717957	G	A
24717973	T	C
24718033	A	G
24718047	C	T
24718301	G	C
24718361	-	C
24718361	T	A
24718373	T	G
24718507	C	G
24718509	A	C
24718524	T	C
24718537	G	A
24718628	C	T
24718714	A	G
24718779	T	C
24718958	G	A
24719011	A	G
24719052	T	C
24719077	G	A
24719089	T	C
24719092	A	G
24719252	T	A
24719895	A	T
24720023	CAGA	-
24720327	T	-
24720389	G	C
24720638	T	C
24720665	C	-
24720671	G	C
24720710	A	T
24720716	C	T
24720748	G	A
24720799	T	A
24720855	G	A
24721382	G	T
24721565	ATA	-
24721680	C	G
24721716	A	-

24721858	CTGAATCCGCAATAT	-
24722036	T	C
24722205	C	T
24722804	G	A
