# Supplementary material for: Beyond the MHC: A canine model of dermatomyositis shows a complex pattern of genetic risk involving novel loci
Source: PLoS Genet. 2017 Feb 3;13(2):e1006604. doi: 10.1371/journal.pgen.1006604 (PMC5315411; doi:10.1371/journal.pgen.1006604)
Supplement: S4 Table — (PDF) [file pgen.1006604.s010.pdf]

S4 Table. Frequency of *PAN2* and *MAP3K7CL* genotypes.

Genotype	Cases (n=132)	Controls (n=390)	OR	95%CI	*P*-value
AA*	84	47	12.77	8.001-20.39	$1.81X10^{-29}$
Aa	40	175	0.53	0.35-0.81	0.0041
aa	8	168	0.085	0.041-0.18	$5.40X10^{-15}$
**No. of *A* alleles (2n)**	208	269	7.042	5.025-10	$2.08X10^{-35}$
BB†	68	23	16.95	9.86-29.16	$8.26X10^{-29}$
Bb	29	126	0.59	0.37-0.94	0.0274
bb	35	241	0.22	0.14-0.35	$1.61X10^{-12}$
**No. of *B* alleles (2n)**	165	172	5.88	4.31-8.065	$1.45X10^{-33}$

**A*=the *PAN2* variant encoding p.Arg492Cys; *a*=wild type

†*B*=*MAP3K7CL* c.383_392ACTCCACAAA>GACT; *b*=wild type
