# Supplementary material for: Beyond the MHC: A canine model of dermatomyositis shows a complex pattern of genetic risk involving novel loci
Source: PLoS Genet. 2017 Feb 3;13(2):e1006604. doi: 10.1371/journal.pgen.1006604 (PMC5315411; doi:10.1371/journal.pgen.1006604)
Supplement: S5 Table — (PDF) [file pgen.1006604.s011.pdf]

S5 Table. Three-locus genotypes for 229 individuals of 30 breeds.

	BREED	GENOTYPE		
		PAN2 R492C	*MAP3K7CL* indel	*DLA-DRB1*
1	AMERICAN BULLDOG	GG	wt	*001:01/015:01*
2	AMERICAN BULLDOG	GG	wt	*001:01/*uncharacterized
3	AMERICAN BULLDOG	GG	wt	*015:02/015:02*
4	AMERICAN BULLDOG	GG	wt	*018:01/018:01*
5	AMERICAN BULLDOG	GG	wt	*018:01/018:01*
6	AMERICAN ESKIMO	GG	wt	*015:01/015:02*
7	AMERICAN ESKIMO	GG	wt	*015:01/040:01*
8	AMERICAN ESKIMO	AG	wt	*015:01/040:01*
9	AMERICAN ESKIMO	GG	wt	*015:01/040:01*
10	AMERICAN ESKIMO	GG	wt	*013:01/040:01*
11	AUSTRALIAN SHEPHERD	GG	wt	*002:01/046:01*
12	AUSTRALIAN SHEPHERD	GG	indel/wt	*026:01/030:01*
13	AUSTRALIAN SHEPHERD	GG	wt	*006:01/011:01*
14	AUSTRALIAN SHEPHERD	GG	wt	*002:01/011:01*
15	AUSTRALIAN SHEPHERD	GG	wt	*011:01/015:01*
16	AUSTRALIAN SHEPHERD	GG	wt	*001:01/015:01*
17	BASENJI	GG	wt	*009:01/030:01*
18	BASENJI	GG	wt	*009:01/027:01*
19	BASENJI	GG	wt	*004:01/027:01*
20	BASENJI	GG	wt	*009:01/022:01*
21	BASENJI	GG	wt	uncharacterized
22	BORDER COLLIE	GG	wt	*013:01/023:01*
23	BORDER COLLIE	GG	wt	*013:01/013:01*
24	BORDER COLLIE	GG	wt	*015:01/023:01*
25	BORDER COLLIE	GG	wt	*013:01/013:01*
26	BORDER COLLIE	GG	wt	*013:01/018:01*
27	BOSTON TERRIER	GG	wt	*002:0*/015:0**
28	BOSTON TERRIER	GG	wt	*002:01/002:01*
29	BOSTON TERRIER	GG	indel/wt	*002:01/002:01*
30	BOSTON TERRIER	GG	indel/wt	*002:01/002:01*
31	BOSTON TERRIER	GG	indel/wt	*002:01/002:01*
32	BOSTON TERRIER	GG	wt	*002:01/002:01*
33	BOSTON TERRIER	GG	wt	*002:01/002:01*
34	BOSTON TERRIER	GG	wt	*001:01/002:01*
35	BOSTON TERRIER	GG	wt	*002:01/023:01*
36	BOXER	GG	wt	*004:01/015:02*
37	BOXER	GG	wt	*001:01/001:01*
38	BOXER	GG	wt	*015:01/*uncharacterized
39	BOXER	GG	wt	*001:01/020:01*
40	BOXER	GG	wt	*012:01/012:01*

41	CAIRN TERRIER	AA	wt	*009:01/015:02*
42	CAIRN TERRIER	AG	indel/wt	*015:01/015:01*
43	CAIRN TERRIER	AG	wt	*009:01/012:01*
44	CAIRN TERRIER	GG	wt	*015:02/015:02*
45	CAIRN TERRIER	AA	wt	*015:01/015:01*
46	CAIRN TERRIER	AG	wt	*009:01/015:02*
47	CAIRN TERRIER	AA	indel/wt	*015:01/015:02*
48	CAIRN TERRIER	AG	indel/wt	*009:01/015:02*
49	CAIRN TERRIER	AG	wt	*009:01/015:02*
50	CAIRN TERRIER	AA	wt	*015:02/018:01*
51	CAIRN TERRIER	AA	wt	*015:01/015:02*
52	CAIRN TERRIER	AA	indel/wt	*015:01/015:02*
53	CAIRN TERRIER	AG	wt	*001:01/001:01*
54	CAIRN TERRIER	AG	wt	*002:01/015:02*
55	CAIRN TERRIER	AA	indel/wt	*009:01/015:01*
56	CAIRN TERRIER	AA	indel/wt	*001:01/009:01*
57	CAIRN TERRIER	AA	indel/wt	*015:01/015:02*
58	CAIRN TERRIER	AA	wt	*001:01/015:02*
59	CARDIGAN WELSH CORGI	AG	indel/wt	*015:02/015:02*
60	CARDIGAN WELSH CORGI	GG	wt	*015:02/015:02*
61	CARDIGAN WELSH CORGI	GG	indel/wt	*020:01/020:01*
62	CARDIGAN WELSH CORGI	GG	wt	*015:02/015:02*
63	CARDIGAN WELSH CORGI	AG	wt	*002:01/015:02*
64	CARDIGAN WELSH CORGI	GG	wt	*002:01/002:01*
65	CARDIGAN WELSH CORGI	AG	wt	*002:01/015:02*
66	CARDIGAN WELSH CORGI	GG	indel/wt	*002:01/018:01*
67	CARDIGAN WELSH CORGI	AA	indel/wt	*002:01/002:01*
68	CARDIGAN WELSH CORGI	GG	wt	*002:01/015:02*
69	CARDIGAN WELSH CORGI	GG	indel/wt	*002:01/002:01*
70	CARDIGAN WELSH CORGI	GG	wt	*015:02/020:01*
71	CARDIGAN WELSH CORGI	AG	wt	*002:01/002:01*
72	CATAHOULA	GG	wt	*001:01/015:01*
73	CATAHOULA	GG	wt	*001:01/015:01*
74	CATAHOULA	GG	wt	*001:01/001:01*
75	CATAHOULA	GG	wt	*026:01/030:01*
76	CATAHOULA	GG	wt	*026:01/030:01*
77	CAVALIER KING CHARLES SPANIEL	GG	wt	*011:01/011:01*
78	CAVALIER KING CHARLES SPANIEL	GG	wt	*009:01/011:01*
79	CAVALIER KING CHARLES SPANIEL	GG	indel/indel	*011:01/011:01*
80	CAVALIER KING CHARLES SPANIEL	GG	wt	*006:01/006:01*
81	CAVALIER KING CHARLES SPANIEL	GG	indel/indel	*006:01/020:01*
82	CHIHUAHUA	AA	wt	uncharacterized
83	CHIHUAHUA	AG	wt	*002:01/009:01*
84	CHIHUAHUA	AG	wt	*001:01/002:01*
85	CHIHUAHUA	AG	wt	*015:01/015:01*

86	CHIHUAHUA	GG	wt	*001:01/009:01*
87	DACHSHUND	GG	wt	*009:01/015:02*
88	DACHSHUND	GG	wt	*001:01/015:01*
89	DACHSHUND	GG	wt	*002:01/009:01*
90	DACHSHUND	GG	wt	*002:0*/015:0**
91	DACHSHUND	GG	wt	*001:01/009:01*
92	DALMATIAN	GG	wt	*002:01/006:01*
93	DALMATIAN	GG	wt	*006:01/020:01*
94	DALMATIAN	GG	indel/wt	*002:01/020:01*
95	DALMATIAN	GG	wt	*002:01/037:01*
96	DALMATIAN	GG	wt	*002:01/020:01*
97	ENGLISH BULLDOG	GG	wt	*015:01/015:02*
98	ENGLISH BULLDOG	GG	wt	*018:01/018:01*
99	ENGLISH BULLDOG	GG	indel/wt	*013:01/015:02*
100	ENGLISH BULLDOG	GG	wt	*013:01/018:01*
101	ENGLISH BULLDOG	GG	wt	*002:01/015:02*
102	ENGLISH COCKER SPANIEL	GG	indel/indel	*001:01/006:01*
103	ENGLISH COCKER SPANIEL	GG	indel/wt	*001:01/001:01*
104	ENGLISH COCKER SPANIEL	GG	indel/wt	*006:01/006:01*
105	ENGLISH COCKER SPANIEL	GG	indel/indel	*006:01/006:01*
106	ENGLISH COCKER SPANIEL	GG	indel/wt	*006:01/006:01*
107	ENGLISH COCKER SPANIEL	GG	indel/indel	*006:01/006:01*
108	ENGLISH COCKER SPANIEL	GG	indel/wt	*006:01/006:01*
109	ENGLISH COCKER SPANIEL	GG	indel/indel	*006:01/006:01*
110	ENGLISH SETTER	GG	wt	*001:01/001:01*
111	ENGLISH SETTER	GG	wt	*001:01/001:01*
112	ENGLISH SETTER	GG	wt	*001:01/001:01*
113	ENGLISH SETTER	GG	wt	*001:01/001:01*
114	ENGLISH SETTER	GG	wt	*001:01/001:01*
115	ENGLISH SPRINGER SPANIEL	GG	indel/wt	*015:01/015:01*
116	ENGLISH SPRINGER SPANIEL	GG	indel/indel	*012:01/015:01*
117	ENGLISH SPRINGER SPANIEL	GG	indel/wt	*015:01/015:01*
118	ENGLISH SPRINGER SPANIEL	GG	wt	*015:01/015:01*
119	ENGLISH SPRINGER SPANIEL	GG	wt	*012:01/012:01*
120	FOX TERRIER	GG	wt	*001:01/015:01*
121	FOX TERRIER	AG	wt	*013:01/013:01*
122	FOX TERRIER	GG	wt	*001:01/001:01*
123	FOX TERRIER	AG	indel/wt	*013:01/013:01*
124	FOX TERRIER	GG	wt	*001:01/001:01*
125	FOX TERRIER	GG	wt	*001:01/001:01*
126	FOX TERRIER	GG	wt	*001:01/013:01*
127	FOX TERRIER	AG	wt	*001:01/001:01*
128	FOX TERRIER	AG	indel/wt	*013:01/013:01*
129	FOX TERRIER	GG	wt	*001:01/001:01*
130	FOX TERRIER	AA	wt	*026:01/030:01*

131	FOX TERRIER	GG	wt	*001:01/001:01*
132	FOX TERRIER	AA	indel/wt	*013:01/013:01*
133	FOX TERRIER	GG	wt	*001:01/018:01*
134	FOX TERRIER	AG	wt	*013:01/013:01*
135	FOX TERRIER	GG	indel/wt	*013:01/013:01*
136	FOX TERRIER	GG	wt	*001:01/001:01*
137	FOX TERRIER	GG	wt	*001:01/001:01*
138	FOX TERRIER	AG	wt	*008:01/013:01*
139	FOX TERRIER	GG	wt	*001:01/001:01*
140	FOX TERRIER	GG	wt	*001:01/001:01*
141	FOX TERRIER	GG	wt	*001:01/001:01*
142	FOX TERRIER	AG	wt	*001:01/001:01*
143	FOX TERRIER	GG	wt	*001:01/015:02*
144	FOX TERRIER	GG	wt	*001:01/018:01*
145	FOX TERRIER	GG	wt	*001:01/001:01*
146	FOX TERRIER	GG	wt	*001:01/013:01*
147	GERMAN SHEPHERD DOG	GG	wt	*001:01/002:01*
148	GERMAN SHEPHERD DOG	GG	wt	*001:02/015:01*
149	GERMAN SHEPHERD DOG	GG	wt	*001:01/011:01*
150	GERMAN SHEPHERD DOG	GG	wt	*001:02/015:01*
151	GERMAN SHEPHERD DOG	GG	wt	*011:01/015:01*
152	GOLDEN RETRIEVER	GG	wt	*006:01/012:01*
153	GOLDEN RETRIEVER	GG	wt	*012:01/012:01*
154	GOLDEN RETRIEVER	GG	wt	*006:01/015:01*
155	GOLDEN RETRIEVER	GG	wt	*006:01/012:01*
156	GOLDEN RETRIEVER	GG	wt	*012:01/012:01*
157	GREAT DANE	GG	indel/wt	*001:01/006:01*
158	GREAT DANE	GG	wt	*001:01/001:01*
159	GREAT DANE	GG	wt	*001:01/012:01*
160	GREAT DANE	GG	wt	*001:01/012:01*
161	GREAT DANE	GG	wt	*001:01/001:01*
162	IRISH SETTER	GG	wt	*001:01/008:01*
163	IRISH SETTER	GG	wt	*001:01/001:01*
164	IRISH SETTER	GG	wt	*001:01/005:01*
165	IRISH SETTER	GG	indel/indel	*001:01/005:01*
166	IRISH SETTER	GG	wt	*005:01/006:01*
167	JACK RUSSELL TERRIER	AA	wt	*015:01/015:02*
168	JACK RUSSELL TERRIER	AG	wt	*002:01/008:01*
169	JACK RUSSELL TERRIER	AA	wt	*001:01/002:01*
170	JACK RUSSELL TERRIER	AA	wt	*011:01/023:01*
171	JACK RUSSELL TERRIER	AG	wt	*009:01/009:01*
172	JACK RUSSELL TERRIER	AA	wt	*002:01/015:02*
173	JACK RUSSELL TERRIER	GG	wt	*013:01/020:01*
174	JACK RUSSELL TERRIER	AA	wt	*002:01/009:01*
175	JACK RUSSELL TERRIER	AG	wt	*011:01/038:01*

176	JACK RUSSELL TERRIER	AG	wt	*013:01/015:01*
177	JACK RUSSELL TERRIER	GG	wt	*006:01/011:01*
178	JACK RUSSELL TERRIER	GG	wt	*015:01/020:01*
179	JACK RUSSELL TERRIER	GG	wt	*009:01/009:01*
180	JACK RUSSELL TERRIER	AG	wt	*013:01/015:01*
181	LABRADOR RETRIEVER	GG	wt	*006:01/012:01*
182	LABRADOR RETRIEVER	GG	wt	*008:02/015:02*
183	LABRADOR RETRIEVER	GG	wt	*001:01/012:01*
184	LABRADOR RETRIEVER	GG	indel/indel	*008:02/012:01*
185	LABRADOR RETRIEVER	GG	wt	*012:01/015:02*
186	LABRADOR RETRIEVER	GG	indel/wt	*012:01/012:01*
187	LABRADOR RETRIEVER	GG	indel/wt	*012:01/012:01*
188	LABRADOR RETRIEVER	GG	indel/wt	*002:01/046:01*
189	LABRADOR RETRIEVER	GG	wt	*001:01/012:01*
190	LABRADOR RETRIEVER	GG	wt	*008:02/020:01*
191	LABRADOR RETRIEVER	GG	wt	*015:02/015:02*
192	LABRADOR RETRIEVER	GG	wt	*012:01/015:02*
193	LABRADOR RETRIEVER	GG	wt	*008:02/020:01*
194	LABRADOR RETRIEVER	GG	wt	*012:01/015:02*
195	PEMBROKE WELSH CORGI	AG	wt	*012:01/015:01*
196	PEMBROKE WELSH CORGI	AG	wt	*012:01/015:01*
197	PEMBROKE WELSH CORGI	AG	wt	*002:01/002:01*
198	PEMBROKE WELSH CORGI	AG	wt	*012:01/015:01*
199	PEMBROKE WELSH CORGI	GG	wt	*012:01/018:01*
200	PEMBROKE WELSH CORGI	GG	wt	*002:01/015:01*
201	PEMBROKE WELSH CORGI	GG	wt	*002:01/015:01*
202	PEMBROKE WELSH CORGI	AG	wt	*002:01/015:01*
203	PEMBROKE WELSH CORGI	AG	wt	*012:01/015:01*
204	PEMBROKE WELSH CORGI	AG	wt	*012:01/015:01*
205	POODLE	GG	wt	*001:01/015:01*
206	POODLE	GG	wt	*015:01/015:01*
207	POODLE	GG	wt	*015:01/015:01*
208	POODLE	GG	wt	*015:01/015:01*
209	POODLE	GG	indel/wt	*015:01/020:01*
210	POODLE	GG	wt	*015:01/015:01*
211	SCHNAUZER	GG	wt	*001:01/013:01*
212	SCHNAUZER	GG	wt	*009:01/009:01*
213	SCHNAUZER	GG	wt	*001:01/009:01*
214	SCHNAUZER	GG	indel/wt	*009:01/015:02*
215	SCHNAUZER	GG	wt	*009:01/009:01*
216	WEST HIGHLAND WHITE TERRIER	AG	wt	*001:01/015:02*
217	WEST HIGHLAND WHITE TERRIER	AA	wt	*001:01/015:01*
218	WEST HIGHLAND WHITE TERRIER	AG	wt	*001:01/001:01*
219	WEST HIGHLAND WHITE TERRIER	GG	wt	*001:01/015:01*
220	WEST HIGHLAND WHITE TERRIER	GG	wt	*001:01/015:01*

221	YORKSHIRE TERRIER	AA	wt	*006:01/006:01*
222	YORKSHIRE TERRIER	AG	wt	*006:01/015:02*
223	YORKSHIRE TERRIER	AG	wt	*006:01/006:01*
224	YORKSHIRE TERRIER	AA	wt	*006:01/006:01*
225	YORKSHIRE TERRIER	GG	wt	*006:01/015:02*
226	YORKSHIRE TERRIER	AG	wt	*015:02/015:02*
227	YORKSHIRE TERRIER	AG	wt	*006:01/006:01*
228	YORKSHIRE TERRIER	AG	wt	*015:02/015:02*
229	YORKSHIRE TERRIER	GG	wt	*015:02/015:02*
