# Supplementary material for: Beyond the MHC: A canine model of dermatomyositis shows a complex pattern of genetic risk involving novel loci
Source: PLoS Genet. 2017 Feb 3;13(2):e1006604. doi: 10.1371/journal.pgen.1006604 (PMC5315411; doi:10.1371/journal.pgen.1006604)
Supplement: S6 Table — (PDF) [file pgen.1006604.s012.pdf]

S6 Table. Primers and genotyping method for variants.

Chr	Position	Gene	Forward primer (5′-3′)	Reverse primer (5′-3′)	Genotyping method
10	565958	*ANKRD52*	GGAGTCTGGGTAGTGAGCCT	GATGCCTATGGACAGTGAGTG	Restriction digest: *BamHI*
10	627760	*PAN2*	AAGAAGGACCAAGGCAGTAGG	ACCGACTCAAGGAATCAGACAG	Restriction digest: *AciI*
10	1239562	*STAT6*	CCTCTTCACCACCACTGCTC	CGTAGTTCCTGCCTCTCACC	Sanger sequencing
31	24132273	*MAP3K7CL*	TGGAGAGGCAAGGAAAGGA	CAACCAAGAACATCTGTCGG	Restriction digest: *Hpy188I*
31	24132343	*MAP3K7CL*	CTGGAATAGACACAGACACCGA	CCTAAACCGCCTTGCCTT	Sanger sequencing
